# Supplementary material for: Experimental demonstration of third-order memristor-based artificial sensory nervous system for neuro-inspired robotics
Source: Nat Commun. 2025 Jul 1;16:5754. doi: 10.1038/s41467-025-60818-x (PMC12215477; doi:10.1038/s41467-025-60818-x)
Supplement: Supplementary file 1 — Supplementary Information [file 41467_2025_60818_MOESM1_ESM.pdf]

Supplementary Information

Experimental Demonstration of Third-Order Memristor-based Artificial Sensory Nervous System for Neuro-Inspired Robotics

See-On Park[1], Hakcheon Jeong[1], Seokho Seo[1], Youna Kwon[2], Jongwon Lee[3], and Shinhyun Choi[1,4]**

[1]School of Electrical Engineering, Korea Advanced Institute of Science and Technology (KAIST), Daejeon, Republic of Korea
[2]Nano Convergence Technology Division, National Nanofab Center (NNFC), Daejeon, Republic of Korea
[3]Department of Semiconductor Convergence, Chungnam National University, Daejeon, Republic of Korea
[4]Graduate School of Semiconductor Technology, Korea Advanced Institute of Science and Technology (KAIST), Daejeon, Republic of Korea

* **Address correspondence to** Jongwon Lee and Shinhyun Choi,
Department of Semiconductor Convergence, Chungnam National University, Daejeon, Republic of Korea,
School of Electrical Engineering, Korea Advanced Institute of Science and Technology (KAIST), Daejeon 34141, Republic of Korea,
Email: jwlee80@cnu.ac.kr, and shinhyun@kaist.ac.kr

Table of Contents

1. Supplementary Figures

Supplementary Fig. S1 | Comparisons of the third-order memristor to conventional low-order memristors……………………………………………………………………….5

Supplementary Fig. S2 | Illustrations of unfavorable and favorable habituation characteristics of memristors for memristor-based artificial sensory nervous systems (MASNSs)…………………………………………………………………………..6

Supplementary Fig. S3 | The comparisons among first, second, and third-order memristors…………………………………………………………………...……7

Supplementary Fig. S4 | The optical microscope image of the fabricated third-order memristor………………………………………………………………………...7

Supplementary Fig. S5 | Favorable electrical characteristics of the third-order memristor for large-scale real-world robotic applications..……………………………8

Supplementary Fig. S6 | Robust endurance characteristic of the device………………9

Supplementary Fig. S7 | Yield of the third-order memristor.………………………….9

Supplementary Fig. S8 | *I-V* curves from randomly selected 30 devices within the 8-inch wafer after forming………………………………………………………...10

Supplementary Fig. S9 | Resistive switching and thermal stability in elevated temperatures....…………………………………………………………….……11

Supplementary Fig. S10 | XPS results of the device with and without high temperature process...…………………………………………………….……….…..…12

Supplementary Fig. S11 | Conductance update curve of the device without high temperature process...…………………………………………….…….…..……12

Supplementary Fig. S12 | Non-monotonic conductance updates in potentiation and depression....…………………………..………………………….……………...…13

Supplementary Fig. S13 | Conductance update of the device with various set pulse conditions....……………………………………………………………………….13

Supplementary Fig. S14 | Intrinsic capacitance of the device before and after forming....………………………………………………………………………….14

Supplementary Fig. S15 | Pulsed response of the device with extended pulse intervals....………………………………………………………………………….14

Supplementary Fig. S16 | Stable and non-volatile conductance states of the third-order memristor at habituation.....…………………………………………….……..15

Supplementary Fig. S17 | The frequency-dependent plasticity via the thermal effect of the third-order memristor....…………………………………………………….16

Supplementary Fig. S18 | Pulsed response of the device with consecutive 100 set pulses followed by 100 reset pulses while varying set voltages and temperatures…….17

Supplementary Fig. S19 | Synaptic behaviors associated with habituation…………..18

Supplementary Fig. S20 | Sensitization memory timescale according to the stimulus strength…………………………………………………………………………..19

Supplementary Fig. S21 | Conduction mechanisms of the device in each state……..20

Supplementary Fig. S22 | The conductance update curves of the thin HfO_2 memristor……………………………………….…………………….………..….21

Supplementary Fig. S23 | Conductance increment according to dishabituation stimuli with different pulse orders………………………………………………………22

Supplementary Fig. S24 | Using a heating pulse to adjust the number of set pulses for sensitization…………………………………………………………………...23

Supplementary Fig. S25 | Effects of the negative voltage pulse amplitude on dishabituation…………………………………………………………………24

Supplementary Fig. S26 | Illustrations of the effectiveness of the MASNS for robotic systems…………………………………………………………………………..25

Supplementary Fig. S27 | Experimental setting of the MASNS-implemented robot arm………………………………………………………………………………26

Supplementary Fig. S28 | Operation algorithm of the robot arm system……….…...27

Supplementary Fig. S29 | The conductance curves of the MASNS-implemented robot arm for 500 stimuli………………………………………………………………28

2. Supplementary Tables

Supplementary Table S1 | Comparison with various memristors emulating habituation characteristics…………………………………………………………………...29

Supplementary Table S2 | Effects of set pulse conditions on switching characteristics of the device……………………………………………………………………….30

Supplementary Table S3 | Comparisons of power consumption and processing time of the third-order memristor and circuit-based systems…………………………………30

3. Supplementary Note

Supplementary Note S1 | Synaptic behaviors associated with habituation…………..31

4. Supplementary Movies

Supplementary Movie S1 | The response of the robot arm with a conventional low-order memristor-based MASNS according to the applied stimuli………….(attached file)

Supplementary Movie S2 | The response of the robot arm with the third-order memristor-based MASNS according to the applied stimuli……………….(attached file)

5. Supplementary References……………………………………………………………..34

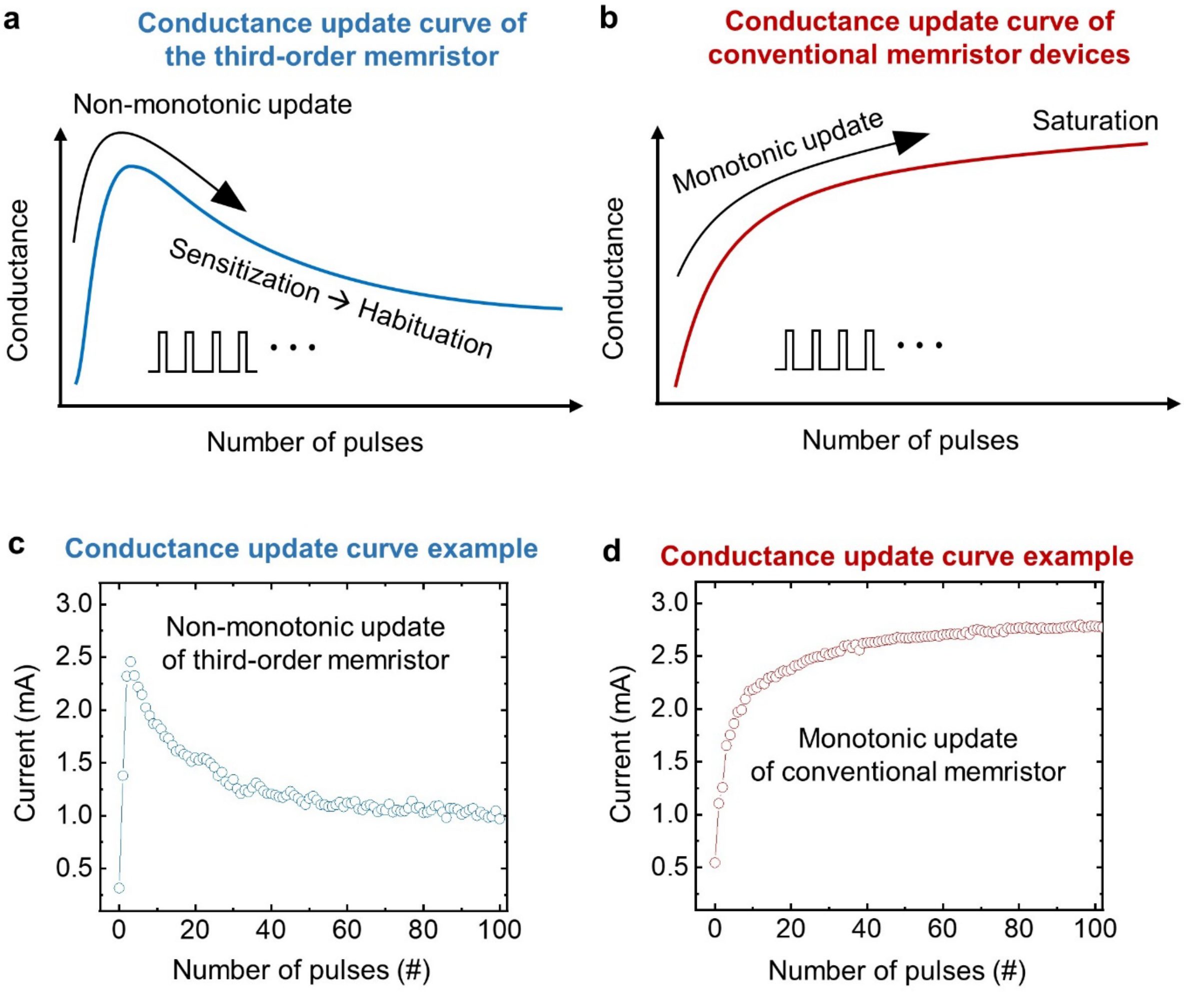

Supplementary Figure S1. Comparisons of the third-order memristor to conventional low-order memristors. a. Illustration of the conductance update curve of the third-order memristor. **b.** Illustration of the conductance update curve of conventional low-order memristors, showing monotonic conductance update curve with conductance saturation. **c.** Example of the conductance update curve from the third-order memristor, demonstrating non-monotonic conductance change. **d.** Example of the conductance update curve from the low-order memristor, showing monotonic conductance update and conductance saturation.

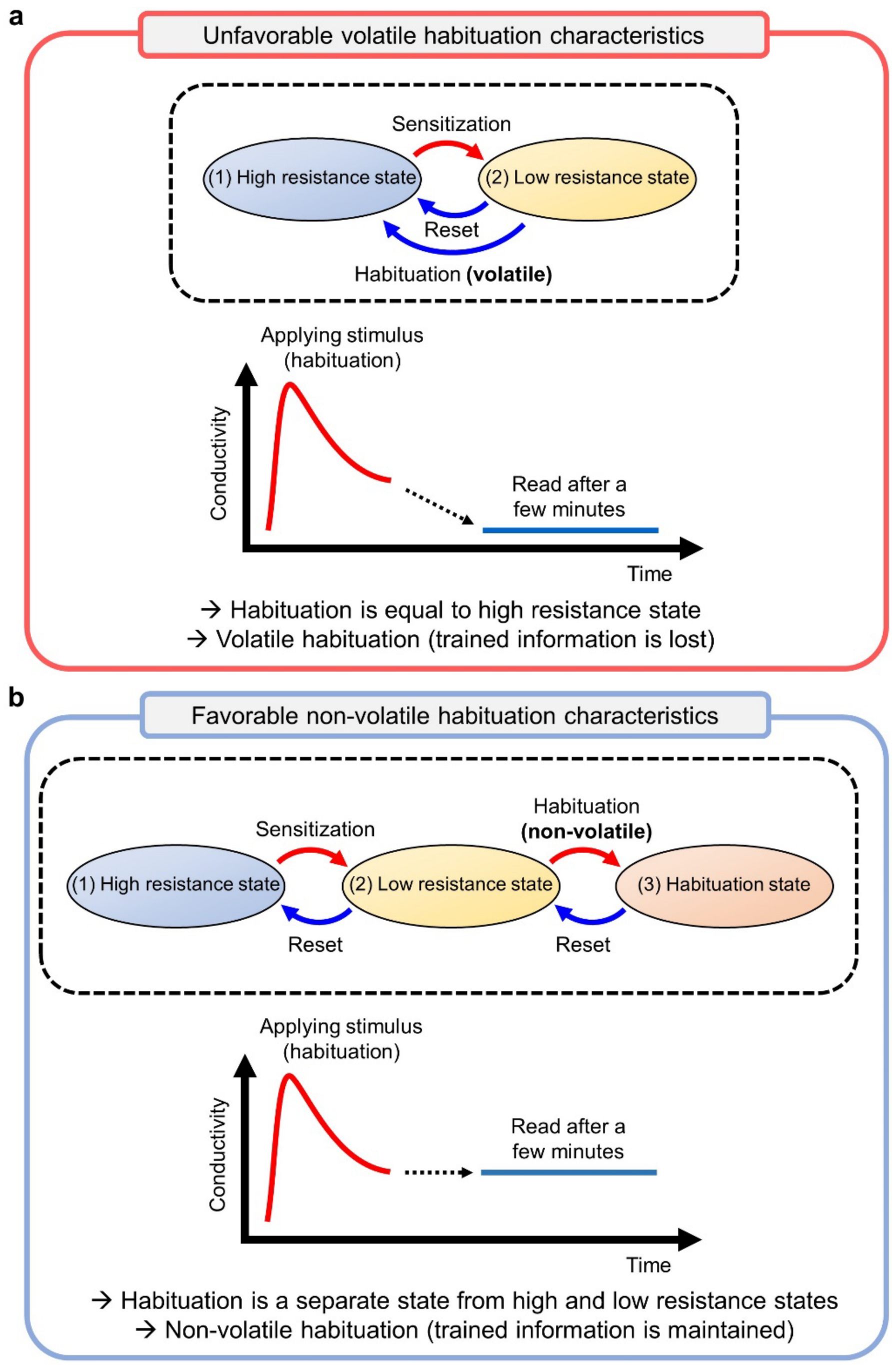

Supplementary Figure S2. Illustrations of unfavorable and favorable habituation characteristics of memristors for memristor-based artificial sensory nervous systems (MASNSs). a. Unfavorable volatile habituation characteristics in various previous studies, where the habituation process is equivalent to the reset process. If the habituation characteristics originate from a filament rupturing due to the Joule heating effect or from a transient ionic diffusion, the habituation state is equivalent to the reset state of the device[1–5]. In those devices with volatile habituation characteristics, the resistance state goes to the reset state after a few minutes, indicating the loss of the trained information. **b.** Favorable non-volatile habituation characteristics, where the habituation is a distinct process from the set or reset process. The habituation state should be non-volatile for a long time so that the trained information can be maintained.

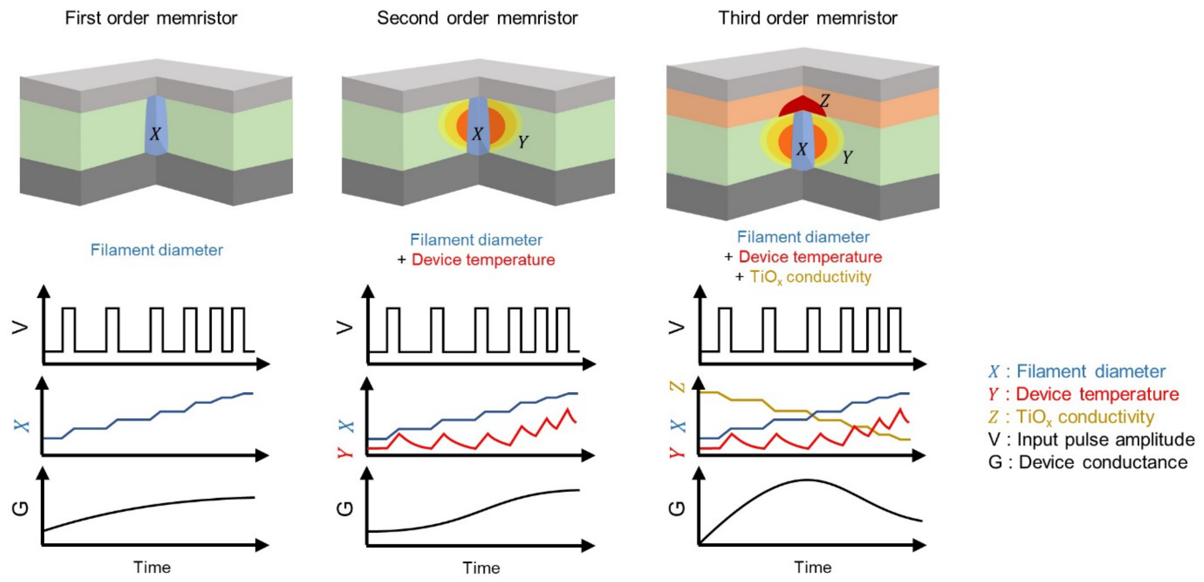

Supplementary Figure S3. The comparisons among first, second, and third-order memristors. *X*, *Y*, and *Z* indicate the filament diameter, the device temperature, and the TiO_x conductivity, respectively. V and G represent the input voltage and device conductance. First-order memristors have only a single state variable, which solely determines the device conductance. Due to their good stability, first-order memristors can be utilized for memory units or vector-matrix-multiplication (VMM) machines[6,7]. However, they lack neuromorphic functions such as sensory nervous system (SNS) functions or time-dependent plasticity. Second-order memristors possess an additional state variable, typically the device temperature. Consequently, the time interval between each input voltage pulse, as well as the input voltage pulse itself, affects the device conductance. Second-order memristors can emulate the time-dependent plasticity of biological synapses and are considered more biologically plausible artificial synapses compared to the first-order memristors[8,9]. However, habituation is difficult to implement in second-order memristors because it requires another state variable that inversely affects the device conductance compared to the filament diameter. Third-order memristors, with three state variables, are expected to exhibit various neuromorphic functions such as habituation, homeostasis, or synaptic reverberation, though only a few have been experimentally demonstrated[10]. In the developed third-order memristor, the TiO_x layer inserted between the Ti electrode and the HfO_2 switching layer provides a third state variable, which affects the device conductance oppositely to the filament and enables the third-order memristor with the habituation function.

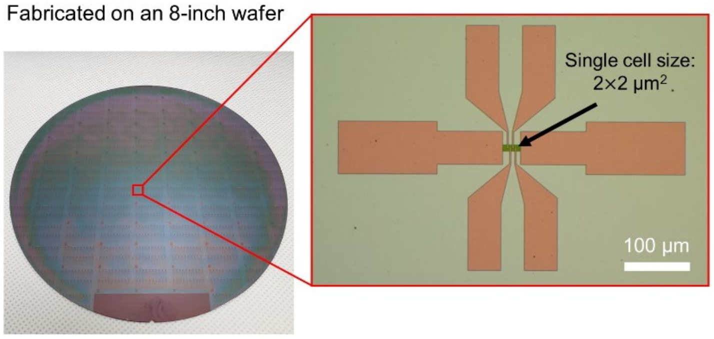

Supplementary Figure S4. The optical microscope image of the fabricated third-order memristor. The third-order memristor arrays are fabricated on an 8-inch wafer through fully CMOS-compatible fabrication processes. The device size for this study was 2×2 μm^2.

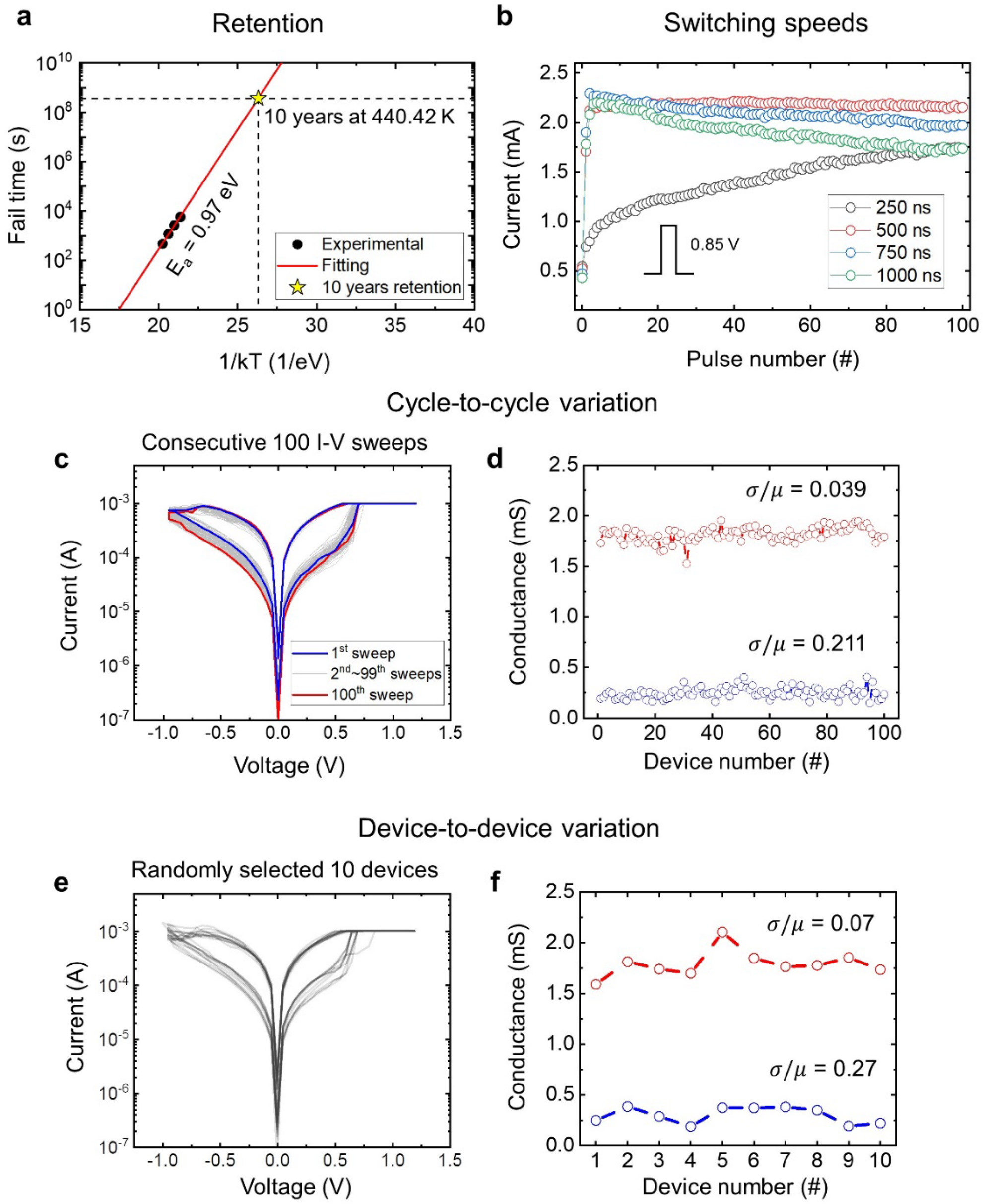

Supplementary Figure S5. Favorable electrical characteristics of the third-order memristor for large-scale real-world robotic applications. a. Retention characteristics of the device showing stable and non-volatile memory with 10 years retention at approximately 440 K. The retention was extracted by monitoring fail time at elevated temperatures (543, 553, 565, and 573 K) and extrapolating the fitted results. The extracted activation energy was 0.97 eV, close to the previously reported activation energy of oxygen vacancies in HfO_2[11]. **b.** Fast switching speeds of the device. The device exhibited abrupt resistive switching and habituation for pulse widths longer than 500 ns. For pulse widths as short as 250 ns, the device displayed resistive switching without habituation. The set pulse amplitude was 0.85 V, and the device was measured by read pulses (0.3 V and 100 μs). **c** and **d.** Cycle-to-cycle variation of the device. Consecutive 100 *I-V* sweeps were applied to the device (**c**). The device exhibited stable and uniform bipolar resistive switching, with a variation coefficient (σ/μ) of 0.039 and 0.211 for LRS and HRS, respectively (**d**). **e** and **f.** Device-to-device variation of the device. The *I-V* curves from randomly selected 10 devices were compared (**e**). The randomly selected devices exhibited similar electrical characteristics without severe device-to-device variation, with a variation coefficient of 0.07 and 0.27 for LRS and HRS, respectively (**f**).

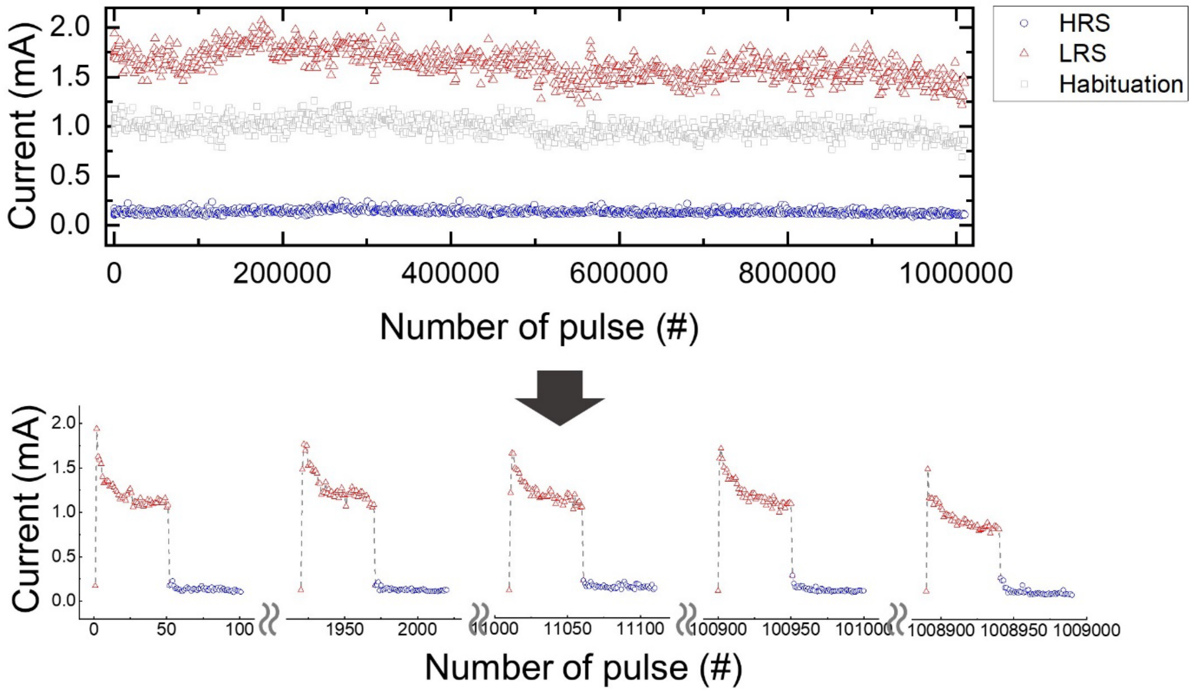

Supplementary Figure S6. Robust endurance characteristic of the device. The endurance characteristic of the device was assessed by applying set and reset pulse trains. Consecutive 50 set pulses (0.75 V and 3 μs) followed by 50 reset pulses (-0.9 V and 10 μs) were repeatedly applied to the device. The total number of set or reset pulses is 10^6. After each set or reset pulse, a read pulse (0.3 V and 100 μs) was applied to measure the device conductance. The device shows good endurance characteristics without significant degradation for habituation characteristics during 10^6 applied pulses.

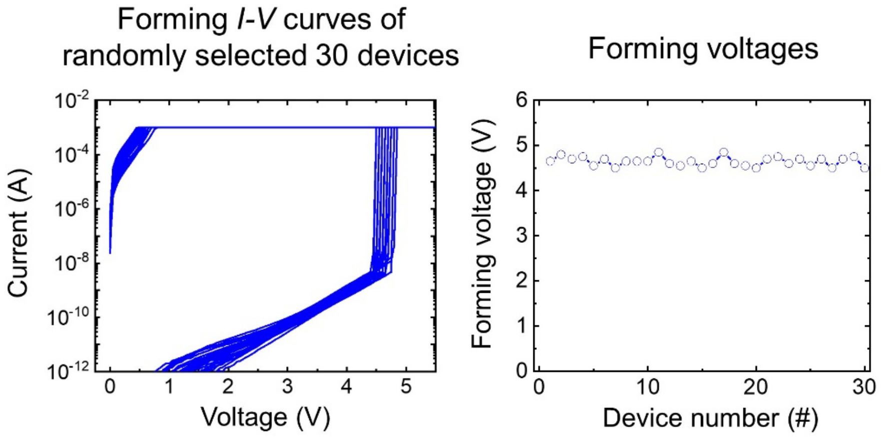

Supplementary Figure S7. Yield of the third-order memristor. a. Forming curves obtained from randomly selected 30 devices within the 8-inch wafer. **b.** Uniform forming voltage distribution of the randomly selected 30 devices, demonstrating great yield with high uniformity of the third-order memristor. The high yield of the device is expected to originate from the mature CMOS-compatible processes with optimized fabrication conditions.

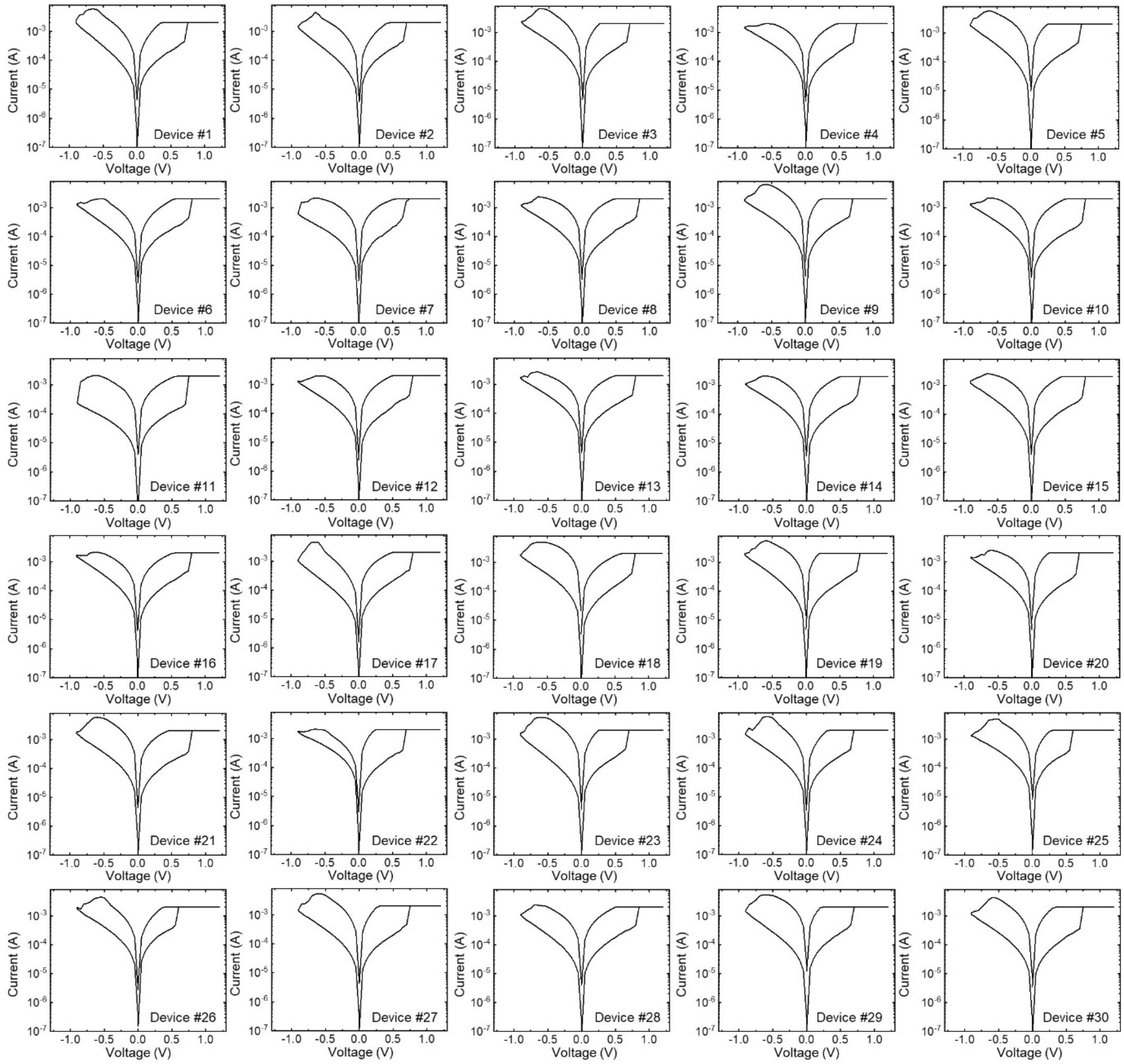

Supplementary Figure S8. *I-V* curves from randomly selected 30 devices within the 8-inch wafer after forming. All the device exhibited similar resistive switching, indicating the high yield of approximately 100%.

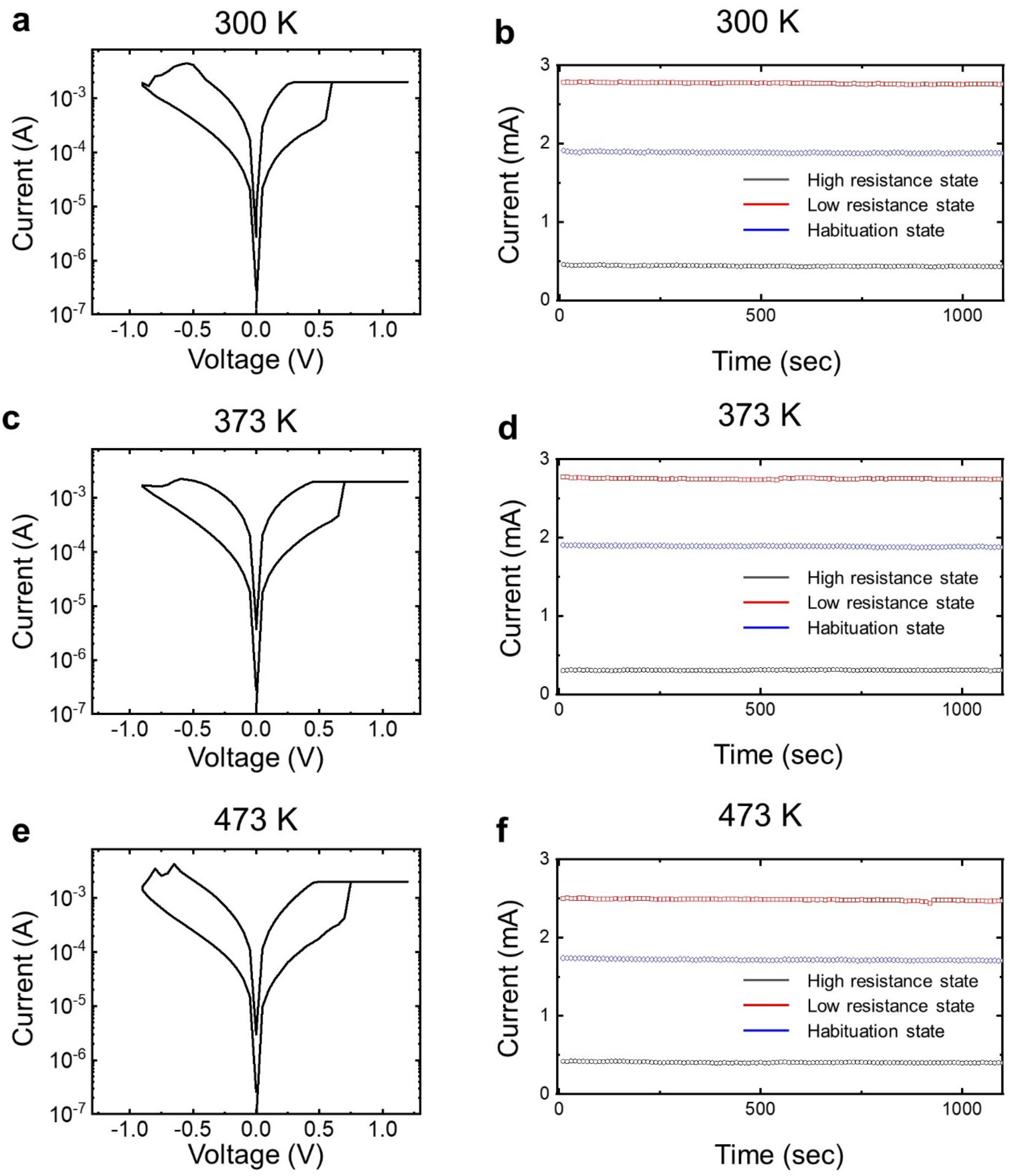

Supplementary Figure S9. Resistive switching and thermal stability in elevated temperatures. a. *I-V* curve of the device at room temperature. **b.** Conductance stability for each state of the device at room temperature. **c.** *I-V* curve of the device at an elevated temperature of 373 K. **d.** Conductance stability for each state of the device at 373 K. **e.** *I-V* curve of the device at an elevated temperature of 473 K. **f.** Conductance stability for each state of the device at 473 K, showing good thermal stability.

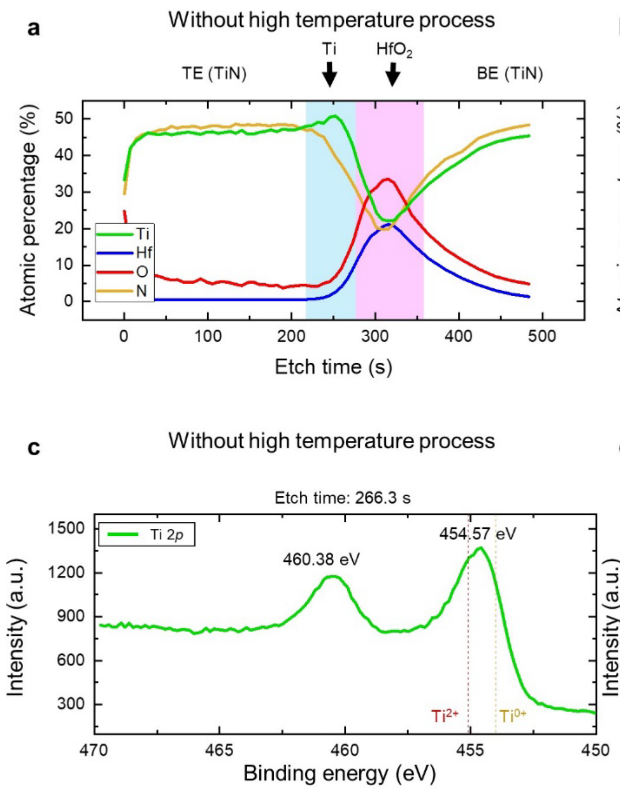

Supplementary Figure S10. XPS results of the device with and without high temperature process. a. Atomic percentage profiles of the device without high temperature process. The depth-profile results show that there is no evident TiO_x layer between the Ti and HfO_2 layers. **b.** Atomic percentage profiles of the device with high temperature process, demonstrating the formation of TiO_x layer between the Ti and HfO_2 layers with a clear shift of oxygen atoms to the Ti layer. **c.** The Ti 2p spectra of the Ti-HfO_2 interface in the device without high temperature process. The peak binding energy of 454.57 eV, which is close to the binding energy of Ti^{0+} (454 eV)[12], demonstrates the metallic Ti is dominant in the Ti-HfO_2 interface without high temperature process. **d.** The Ti 2p spectra of the Ti-HfO_2 interface in the device with high temperature process, showing peak binding energy of 455.06 eV close to that of the Ti^{2+} (455.1 eV)[12] and demonstrating the formation of TiO near the Ti-HfO_2 interface.

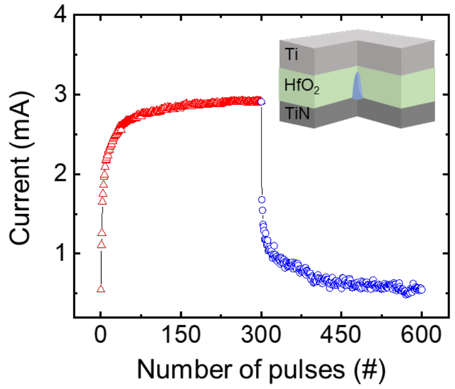

Supplementary Figure S11. Conductance update curve of the device without high temperature process. The device exhibited the monotonic conductance update curve similar to typical low-order memristors. The results demonstrate that the formation of TiO_x layer via the high temperature BEOL process is essential for habituation characteristics.

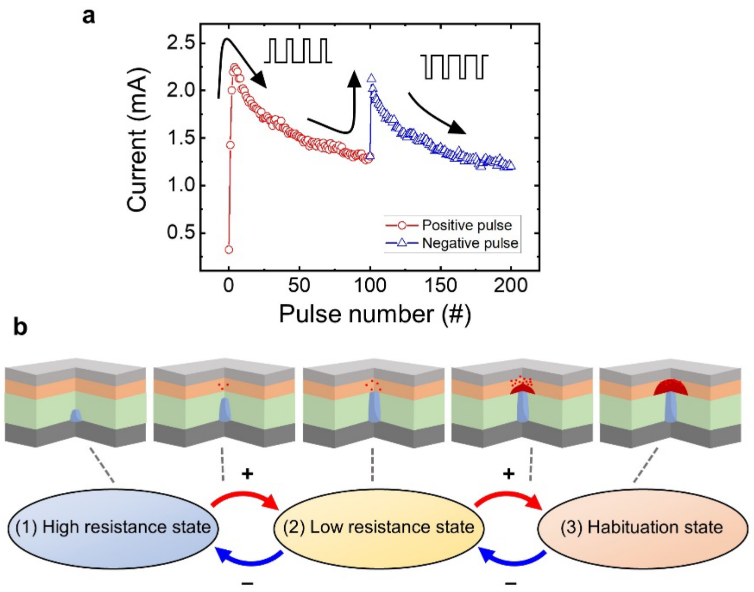

Supplementary Figure S12. Non-monotonic conductance updates in potentiation and depression. a. Conductance update curves with consecutive 100 positive voltage pulses (0.75 V, 5 μs), followed by consecutive 100 negative voltage pulses (-0.8 V, 1 μs). The non-monotonic conductance update properties were observed in depression stage, as well as in potentiation stage. **b.** Illustrations about device switching mechanisms for each state.

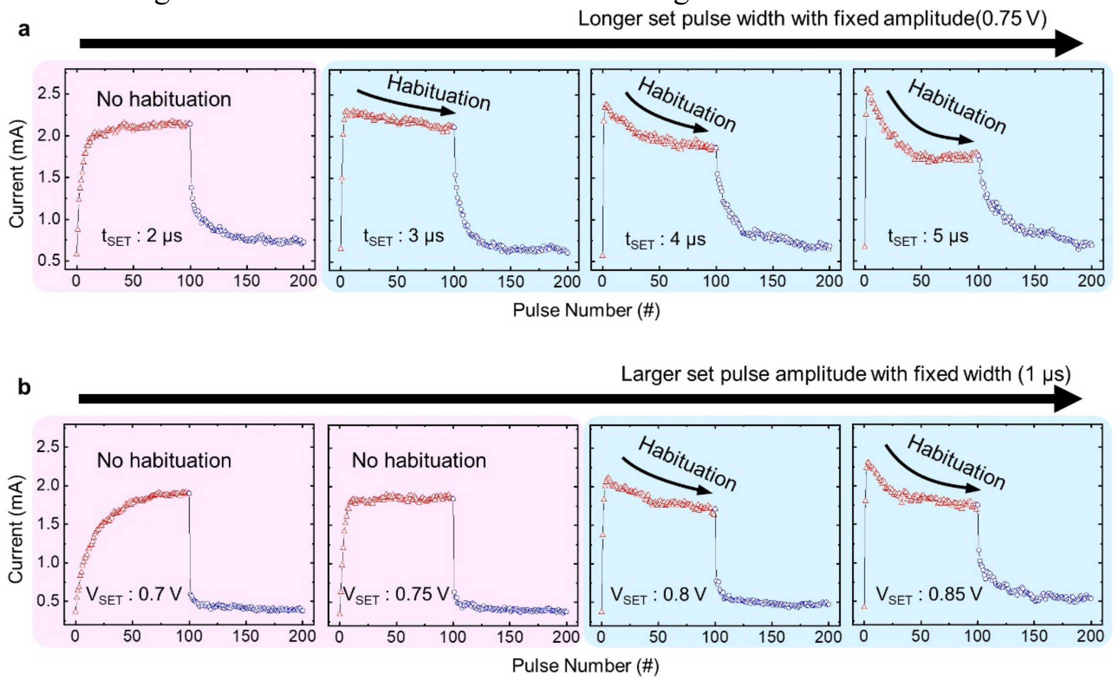

Supplementary Figure S13. Conductance update of the device with various set pulse conditions. a. The conductance update curves of the third-order memristor for consecutive 100 set pulses (0.75 V and 2, 3, 4, and 5 μs, respectively) followed by 100 reset pulses (-0.9 V and 1 μs) are measured. **b.** The conductance update curves of the third-order memristor for consecutive 100 set pulses (0.7, 0.75, 0.8, and 0.85 V and 1 μs, respectively) followed by 100 reset pulses (-0.9 V and 1 μs) are measured. The habituation characteristic becomes evident when the set pulse width is longer (**a**) or when the set pulse amplitude is higher (**b**), demonstrating that enough set pulse amplitude and width are necessary to transfer the oxygen anions into the TiO_x layer. The device conductance is obtained by a read pulse (0.3 V and 50 μs) in both (**a**) and (**b**) after applying each set and reset pulse.

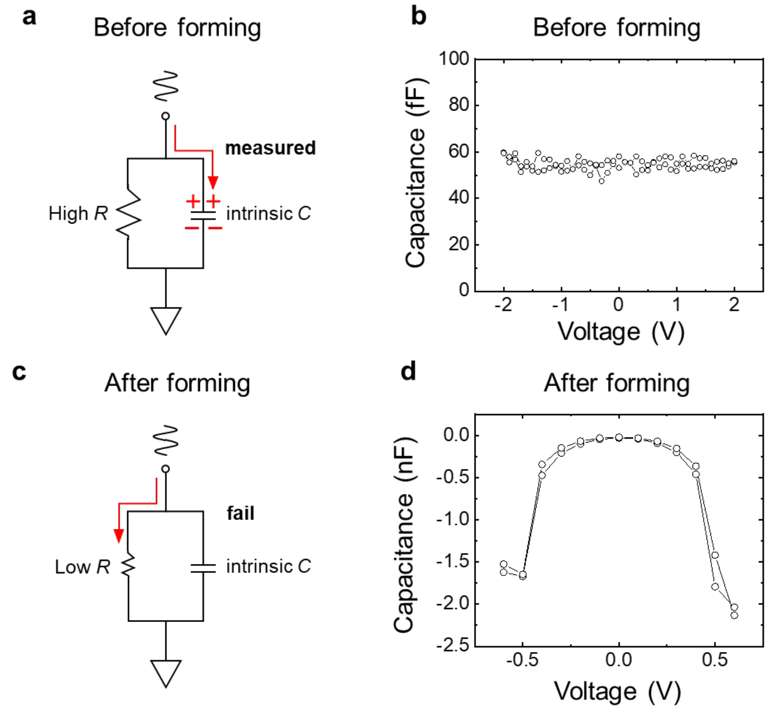

Supplementary Figure S14. Intrinsic capacitance of the device before and after forming. **a.** Schematic of the device with intrinsic capacitance and high electrical resistance before forming. Due to the high electrical resistance, the effect of the intrinsic capacitance could be dominant before forming. **b.** Results of capacitance measurement before forming. **c.** Schematic of the device with intrinsic capacitance and low electrical resistance after forming. Since the electrical resistance after the forming process is small, the effect of the intrinsic capacitance becomes minimal. **d.** Results of capacitance measurement after forming, showing measurement failure due to the low electrical resistance of the device.

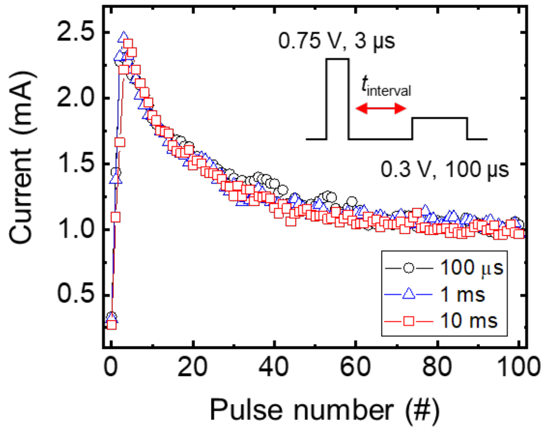

Supplementary Figure S15. Pulsed response of the device with extended pulse intervals. To minimize the possible effect of the current overshoot and *RC* delay, the device was measured by increasing pulse intervals between set pulse and read pulse. Similar output current characteristics for all the pulse interval case demonstrate that the current overshoot and *RC* delay hardly affect the device characteristics.

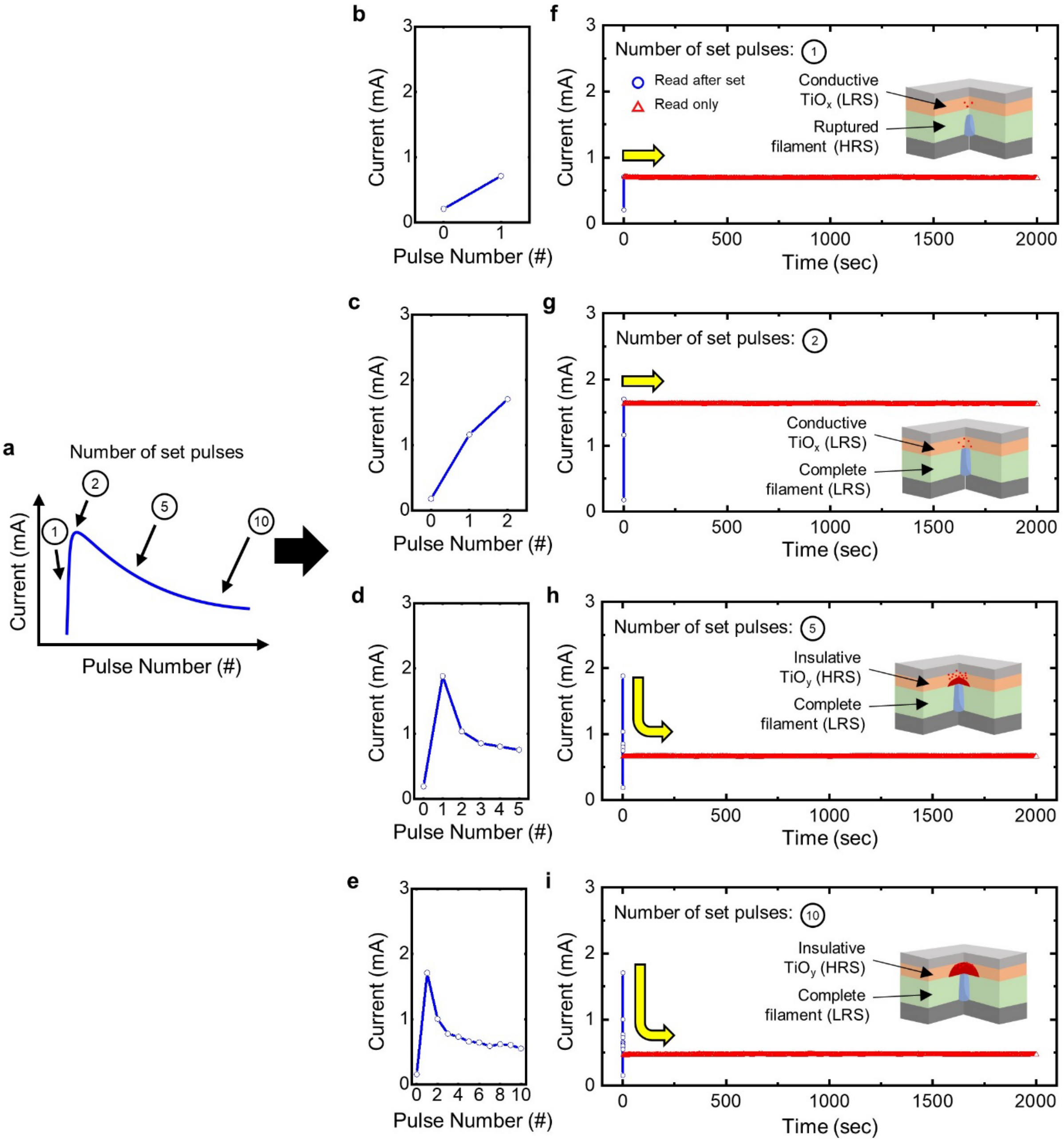

Supplementary Figure S16. Stable and non-volatile conductance states of the third-order memristor at habituation. a. Illustration of the conductance update curve of the third-order memristor according to the number of applied pulses. **b-e.** The device conductance update curve during 1, 2, 5, and 10 set pulses, respectively. **f-i.** The conductance of the device after 1, 2, 5, and 10 set pulses in **b-e** measured over 2,000 seconds. For all cases, the conductance states were stably maintained over 2,000 seconds, demonstrating that every conductance state including the habituation state is stable and non-volatile. Here, a voltage pulse with 0.75 V amplitude and 5 μs width was utilized as the set pulse while a voltage pulse with 0.3 V amplitude and 300 μs width was used as the read pulse.

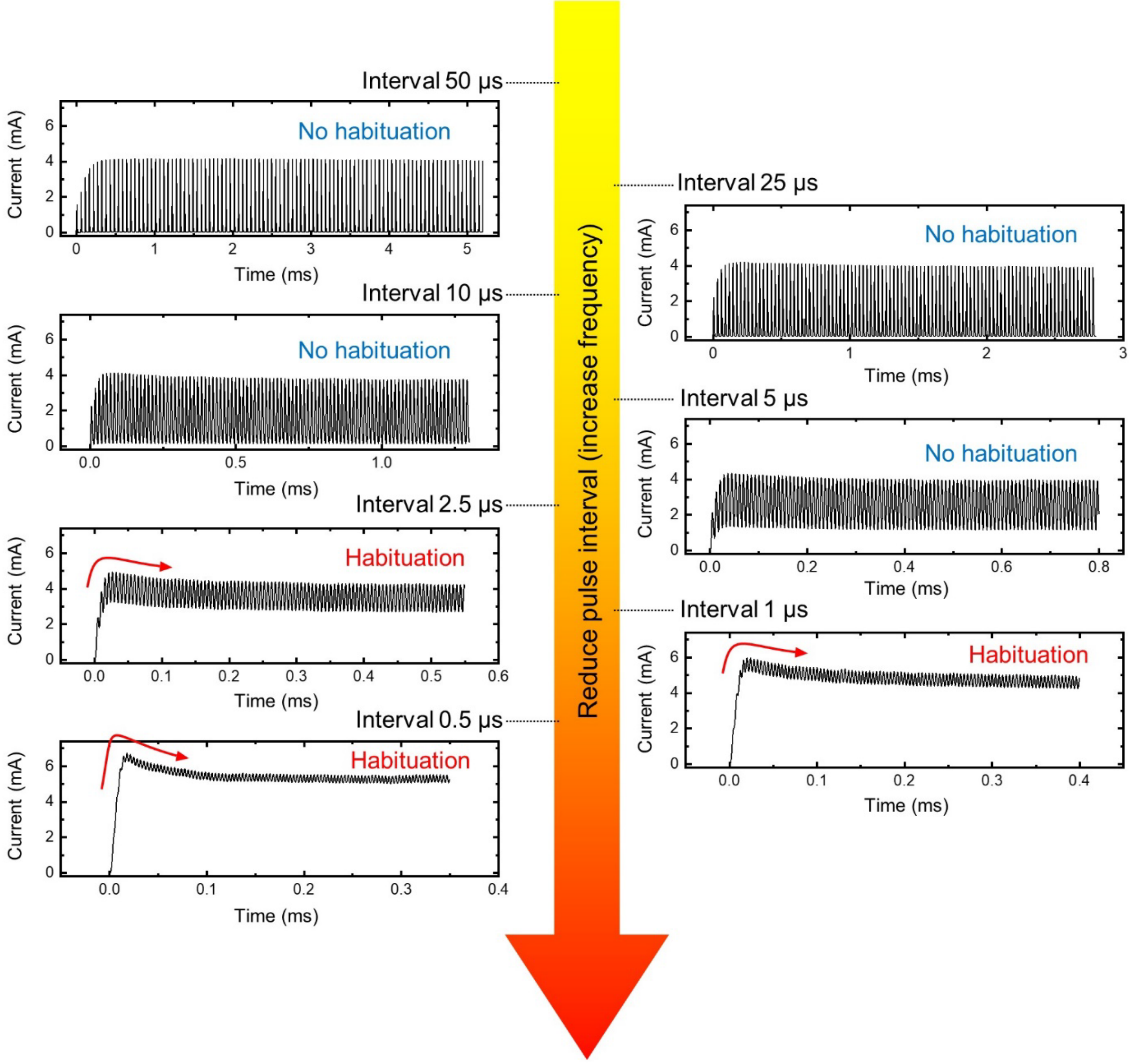

Supplementary Figure S17. The frequency-dependent plasticity via the thermal effect of the third-order memristor. The third-order memristor output current was measured by applying consecutive 100 pulse train (0.75 V and 2 μs) with various pulse intervals (50, 25, 10, 5, 2.5, 1, and 0.5 μs). When the pulse interval was shorter than 2.5 μs, the habituation characteristic was observed. This time-dependent plasticity originates from the thermal effect (second state variable), where the increased device temperature through the Joule heating affects the switching of the device. The results indicate that the device temperature is one of the state variables of the third-order memristor.

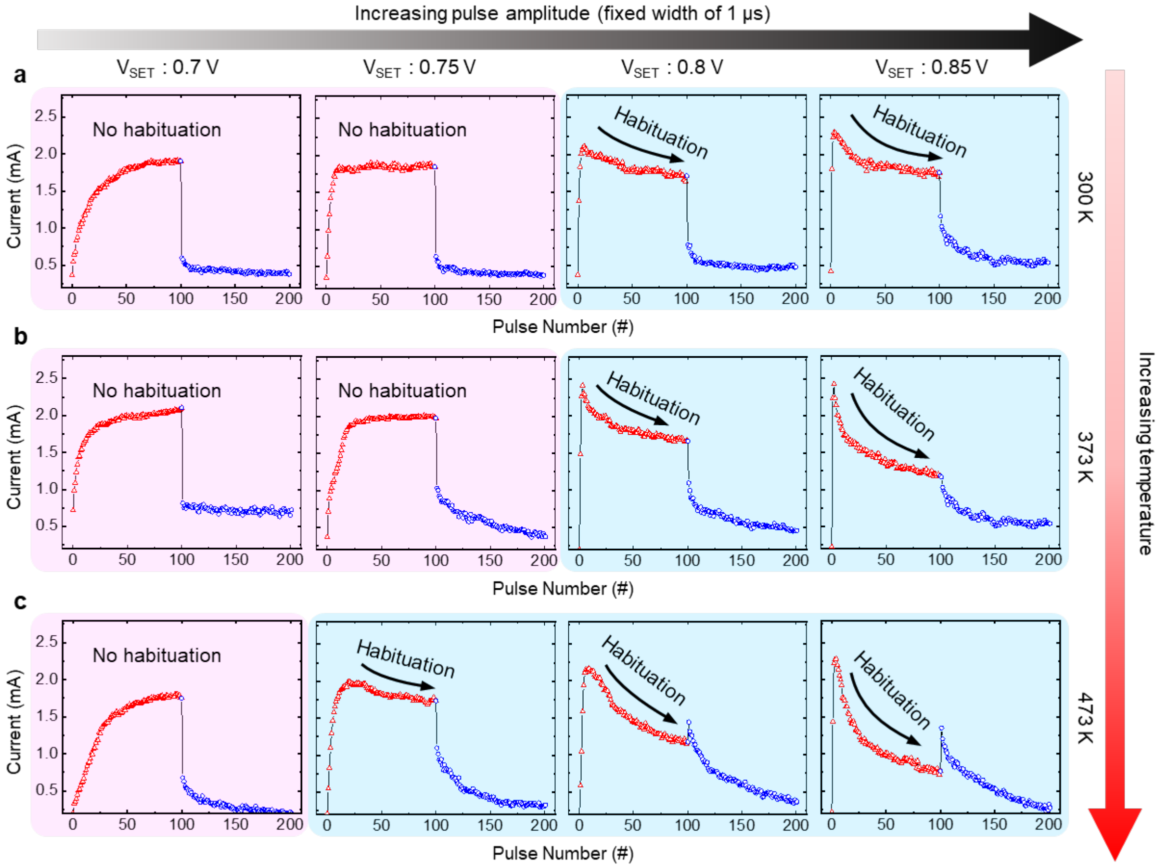

Supplementary Figure S18. Pulsed response of the device with consecutive 100 set pulses followed by 100 reset pulses while varying set voltages and temperatures. a. Pulsed response of the device at room temperature (300 K). Habituation characteristics were observed for the 0.8 V of set pulse amplitude. **b.** Pulsed response of the device at an elevated temperature of 373 K, showing pronounced habituation. **c.** Pulsed response of the device at an elevated temperature of 473 K, showing faster habituation at lower set pulse amplitudes (0.75 V), as well as pronounced habituation.

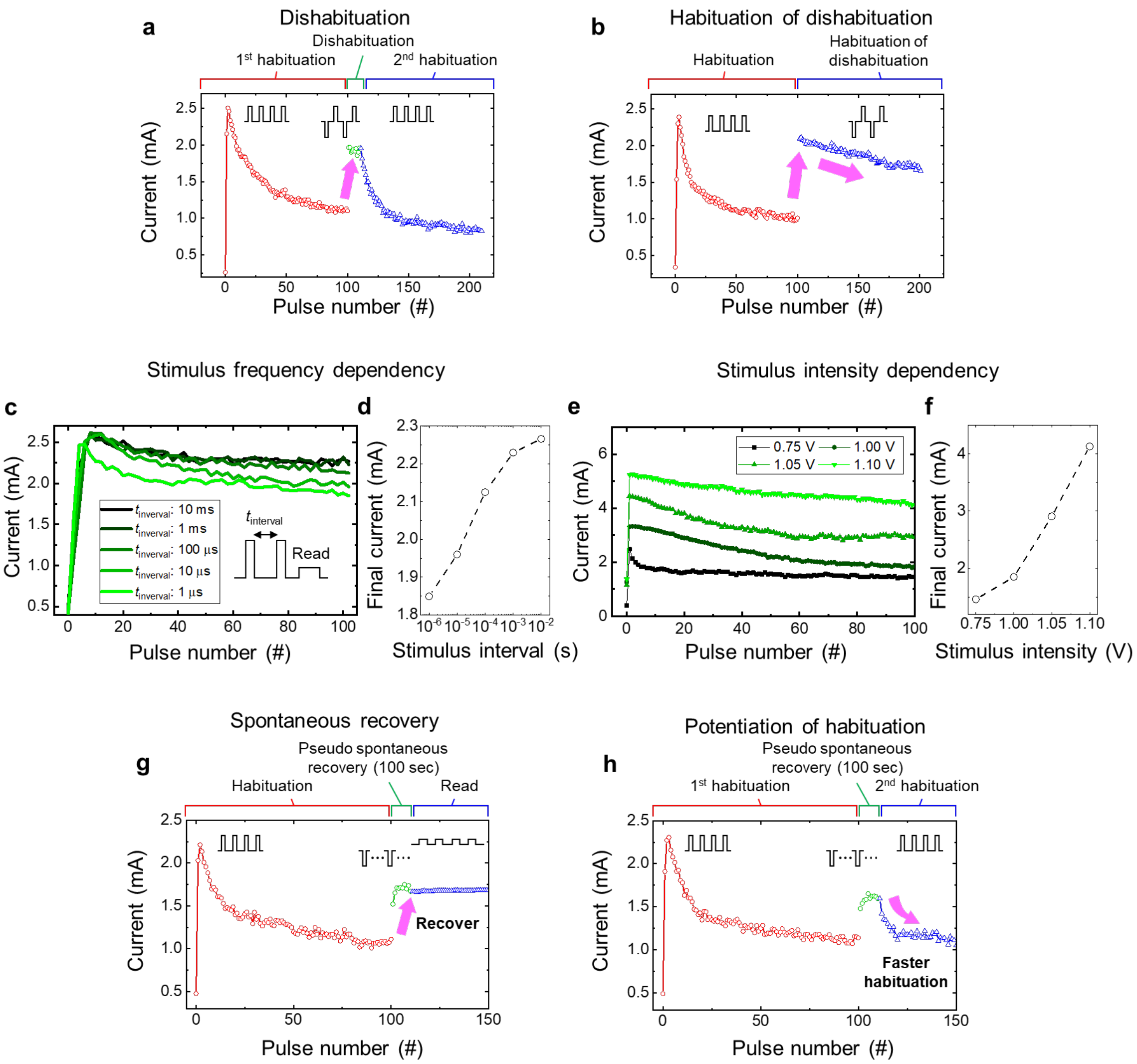

Supplementary Figure S19. Synaptic behaviors associated with habituation. a. Dishabituation. The 100 sets of positive voltage pulses (0.75 V and 5 µs) were applied to the device, resulting in the habituation. After the habituation, consecutive 10 combined negative (-0.9 V and 1 µs) and positive (0.75 V and 5 µs) voltage pulses corresponding to pain and tactile stimuli, respectively, were applied to the device. The presentation of the combined pulses increased the device conductance back to LRS, indicating the dishabituation. **b.** Habituation of dishabituation. The 100 sets of positive voltage pulses (0.75 V and 5 µs) were applied to the device for habituation. After the first habituation, consecutive 100 dishabituation combined voltage pulses (-0.9 V, 1 µs and 0.75 V, 5 µs) were applied to the habituated device. The device conductance initially increased by the dishabituation, but it soon decreased as the dishabituation pulses continued, indicating the habituation against the dishabituation stimuli. **c** and **d.** Stimulus frequency dependency. To investigate the effect of stimuli frequency, two positive voltage pulses (0.75 V, 3 µs) with different pulse intervals (from 1 µs to 10 ms) were repetitively applied to the device (**c**). The device exhibited stimulus frequency dependency with faster habituation and lower final output current for shorter pulse intervals or higher frequencies (**d**). **e** and **f.** Stimulus intensity dependency. Consecutive 100 positive set voltage pulses with different amplitudes of 0.75, 1.00, 1.05, 1.10 V and width of 5 µs were applied to the device (**e**). The device exhibited pronounced habituation for the 0.75 V case. However, the higher pulse amplitude resulted in higher output currents, due to thicker filament formation (**f**). The

results demonstrate the stimulus intensity dependency of the third-order memristor. **g.** Spontaneous recovery. Spontaneous recovery requires time-decaying memory or volatile memory. To emulate spontaneous recovery in the non-volatile third-order memristor, periodic weak reset pulse (-0.7 V, 2 μs) were utilized. After 100 sets of positive voltage pulses (0.75 V, 5 μs) for habituation, positive voltage pulses were withheld for 100 seconds. During this withholding period, the periodic weak reset pulses were applied to the device every 10 seconds. Partial recovery of the habituated state was observed after the 100 seconds of withholding period, demonstrating the pseudo-spontaneous recovery. **h.** Potentiation of habituation. Two stages of habituation events were presented (0.75 V and 5 μs), with the 100 seconds of stimulus withholding period. During the withholding period, the periodic weak reset pulses were applied for pseudo-spontaneous recovery. It was observed that the second habituation was faster than the first habituation, demonstrating the potentiation of habituation. Here, the device output current was measured by the read pulse (0.3 V and 100 μs). The detailed explanations regarding each synaptic behavior are presented in Supplementary Note S1.

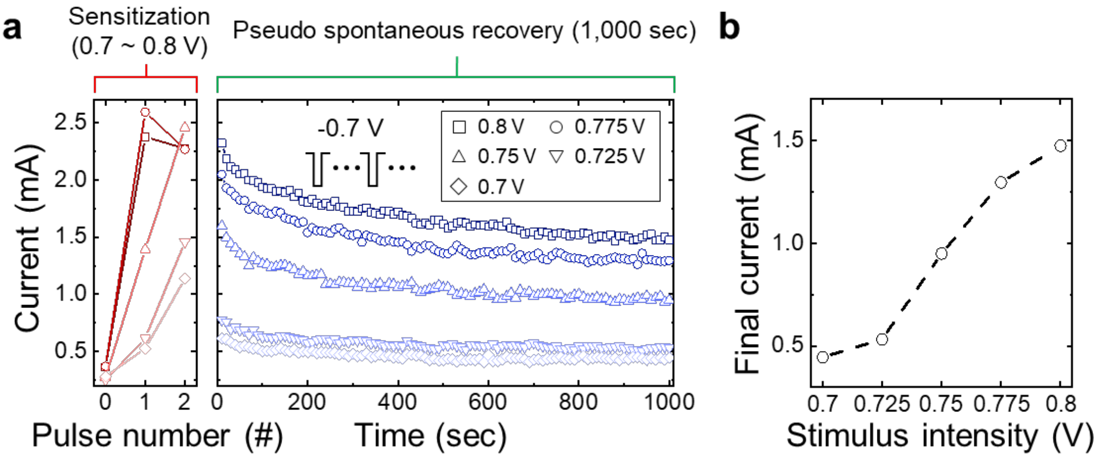

Supplementary Figure S20. Sensitization memory timescale according to the stimulus strength. a. Sensitization memory timescale with pseudo spontaneous recovery. After applying two positive voltage pulses with various amplitudes (0.7, 0.725, 0.75, 0.775, and 0.8 V, 5 μs), stimulus withholding was presented with pseudo spontaneous recovery (-0.7 V and 2 μs per 10 seconds). While periodic weak reset pulse gradually reduced conductance, it was observed that the higher stimulus intensity resulted in higher final conductance after 1,000 seconds. **b.** Output current of the device according to the stimulus intensity after 1,000 seconds of stimulus withholding.

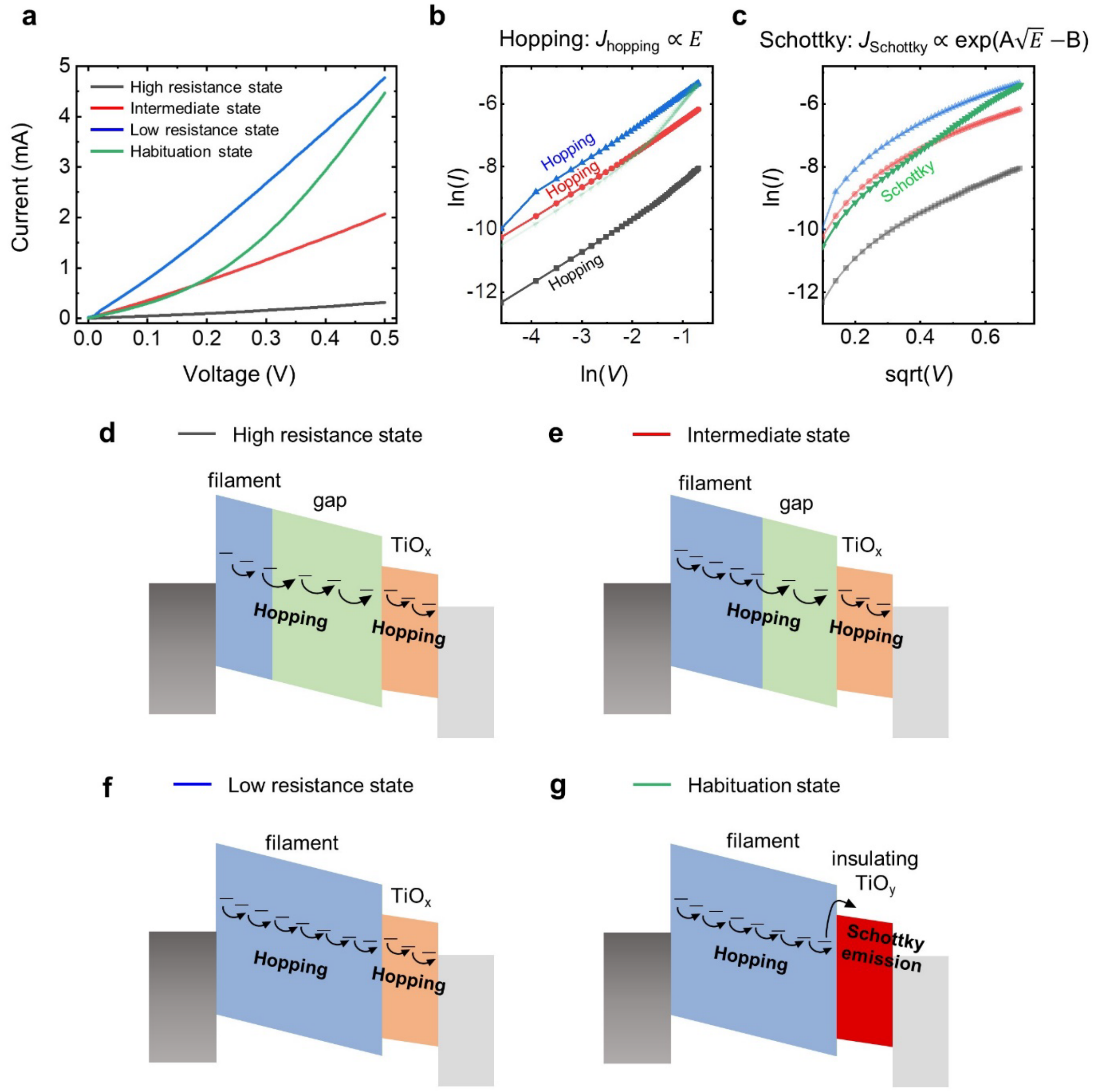

Supplementary Figure S21. Conduction mechanisms of the device in each state. a. *I*-*V* characteristics of the device in HRS, intermediate state between HRS and LRS, LRS, and habituation state. **b.** Hopping conductions in HRS, intermediate state, and LRS state, similar to several oxide-based memristors. **c.** Schottky emission conduction in habituation state, demonstrating that the habituation state has different conduction mechanisms from other states. **d-g.** Switching mechanisms of the device based on the conduction mechanisms of each state. The device in HRS conducts electrons by hopping conduction, where the defect site density varies in the filament and the filament gap (**d**). As the filament growth, the conductivity of the device increases, showing intermediate state between HRS and LRS (**e**). The filament is fully grown in the LRS state, displaying the highest conductivity (**f**). As more set pulses are applied, oxygen anions from the filament to the TiO_x layer oxidize the TiO_x layer, resulting in the formation of an insulating and defectless TiO_y layer (**g**). Due to the lower defect density in the TiO_y layer, the device exhibits the Schottky emission, rather than hopping conduction.

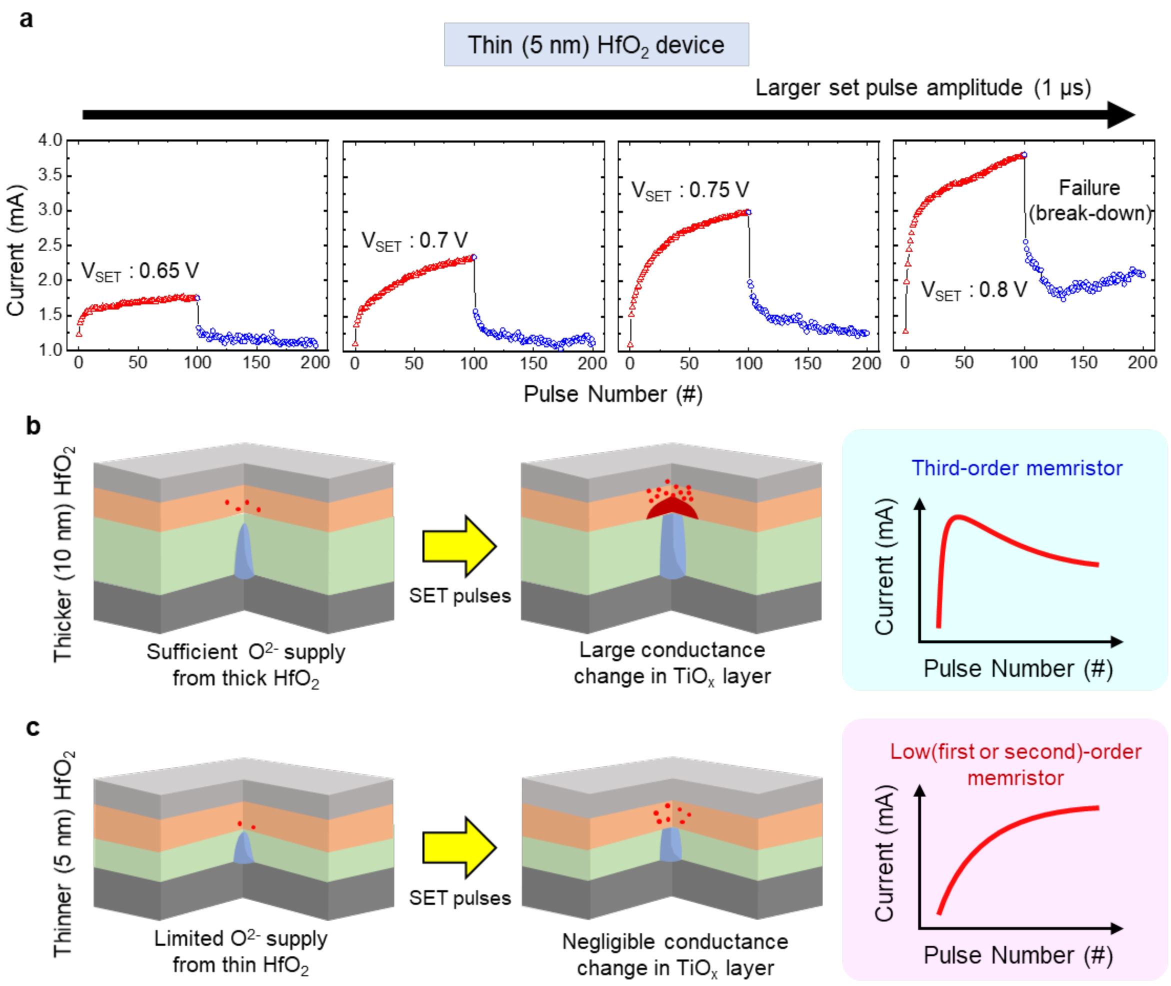

Supplementary Figure S22. The conductance update curves of the thin HfO_2 memristor. **a.** The conductance update curves of the HfO_2 memristor device, having the same device structure as the third-order memristor but having a thinner HfO_2 layer (5 nm), measured with various set pulse voltages (0.65, 0.7, 0.75, and 0.8 V) and fixed reset pulse conditions (-0.8 V, 5 μs). The read pulse of 0.3 V and 50 μs was utilized to measure the conductance of the device. The thinner HfO_2 memristor does not show the habituation characteristic even if the higher voltage pulses are applied and only exhibits the low-order (first- or second-order) characteristic. **b.** Schematic of the device switching and conductance update trend during the set pulses in the device with a thick (10 nm) HfO_2 layer. The device with the thick HfO_2 layer possesses third-order memristor characteristics and habituation function through the oxidation and reduction of the TiO_x layer. **c.** Schematic of the device switching and conductance update trend during the set pulses in the device with a thin (5 nm) HfO_2 layer. Since the thin HfO_2 layer supplies less oxygen to the TiO_x layer, the resistive switching in the TiO_x is negligible, resulting in low-order memristor characteristics.

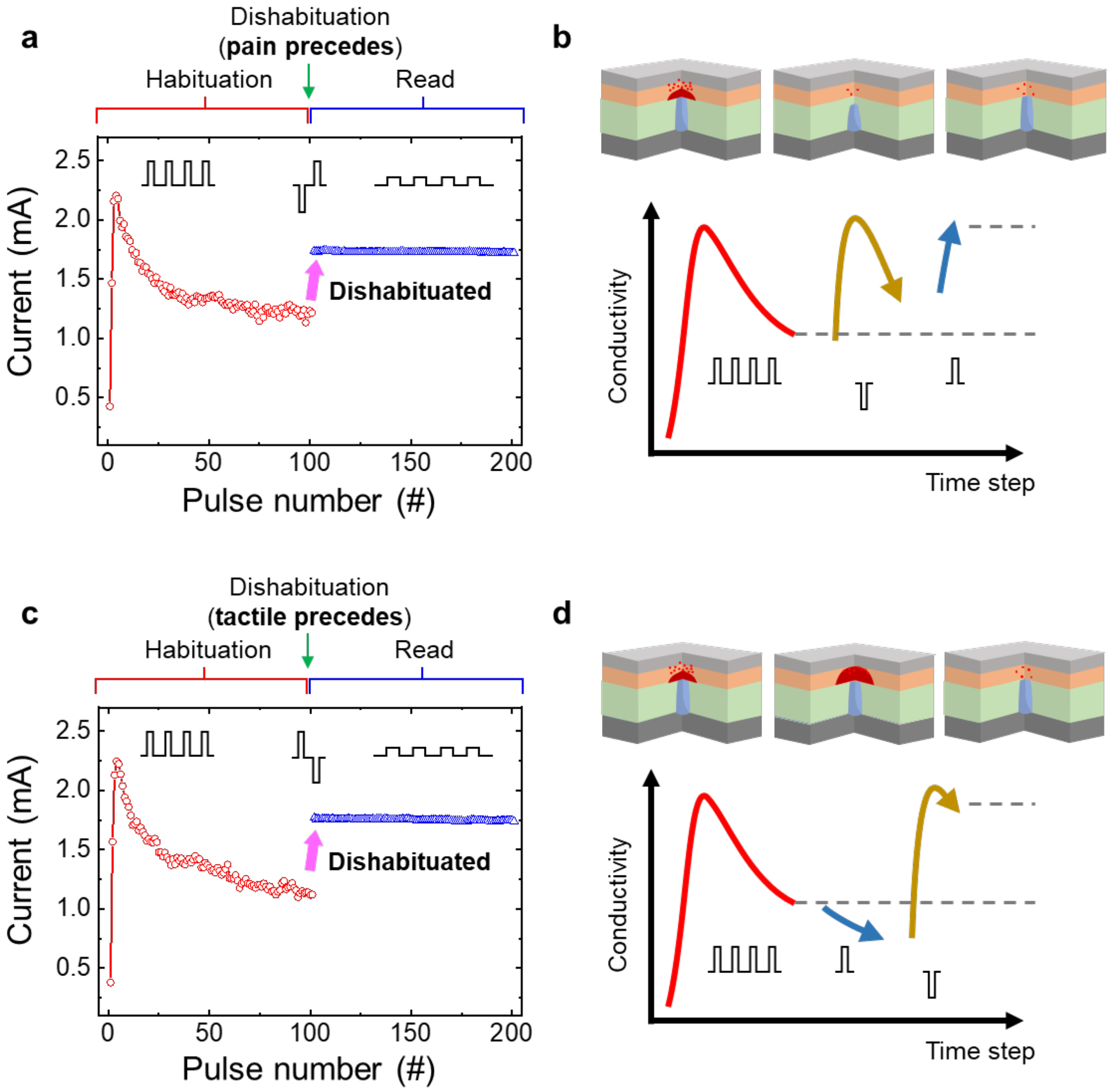

Supplementary Figure S23. Conductance increment according to dishabituation stimuli with different pulse orders. The effects of the stimulus order in dishabituation stimuli were investigated by applying combined voltage pulse with different pulse orders to the habituated device while measuring the conductance change. **a** and **b.** Response of the device when negative voltage pulse precedes. When the negative voltage pulse (-0.9 V, 1 μs) for pain stimulus precedes the positive voltage pulse (0.8 V, 1 μs) for tactile stimulus, dishabituation with increased conductance was observed (**a**). The negative voltage pulse moves oxygen anions in the TiO_x layer into the filament, partially rupturing the filament and resulting in the intermediate state between HRS and LRS. Conversely, the following positive voltage pulse removes oxygen anions in the filament, increasing the device conductance (**b**). **c** and **d.** Response of the device when positive voltage pulse precedes. When the positive voltage pulse for tactile stimulus precedes the negative voltage pulse for pain stimulus, dishabituation with increased conductance was observed (**c**), similar to the reverse order case. The positive voltage pulse moves oxygen anions into the TiO_x layer, making the device more habituated. The following negative voltage pulse relocates oxygen anions from TiO_x layer to the filament, thereby increasing the device conductance (**d**).

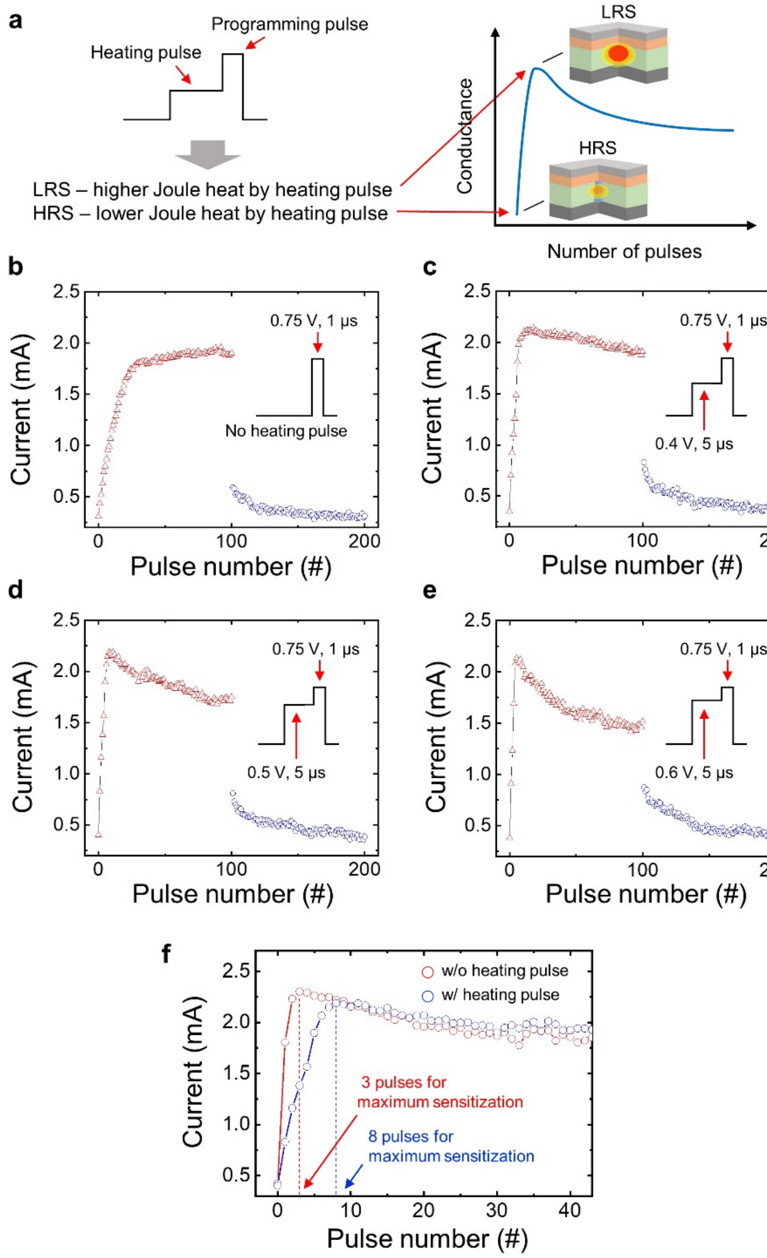

Supplementary Figure S24. Using a heating pulse to adjust the number of set pulses for sensitization. a. Illustration of the heating pulse. A heating pulse is a low voltage pulse that generates Joule heat effect in the device, while not changing the device conductance. **b-e.** Pulsed responses of the device when a heating pulse of 0, 0.4, 0.5, and 0.6 V and 5 μs, respectively, were used. The results without heating pulse exhibited gradual sensitization without habituation characteristics (**b**), while the application of heating pulse induced habituation (**c**, **d**, and **e**). The Joule heat effects from the heating pulse facilitate the oxidation of the TiO_x layer in the device, resulting in habituation characteristics. **f.** Comparison of the habituation characteristics with and without the heating pulse. When the high set voltage pulse (0.85 V and 1 μs) was utilized without the heating pulse, abrupt sensitization was observed, where only three pulses were required for maximum sensitization. On the contrary, when the low set voltage pulse (0.75 V and 1 μs) was used with a heating pulse (0.5 V and 5 μs), more gradual sensitization was observed while showing similar habituation characteristics. The utilization of a heating pulse can be advantageous for adjusting the number of stimuli for sensitization without changing the habituation characteristics.

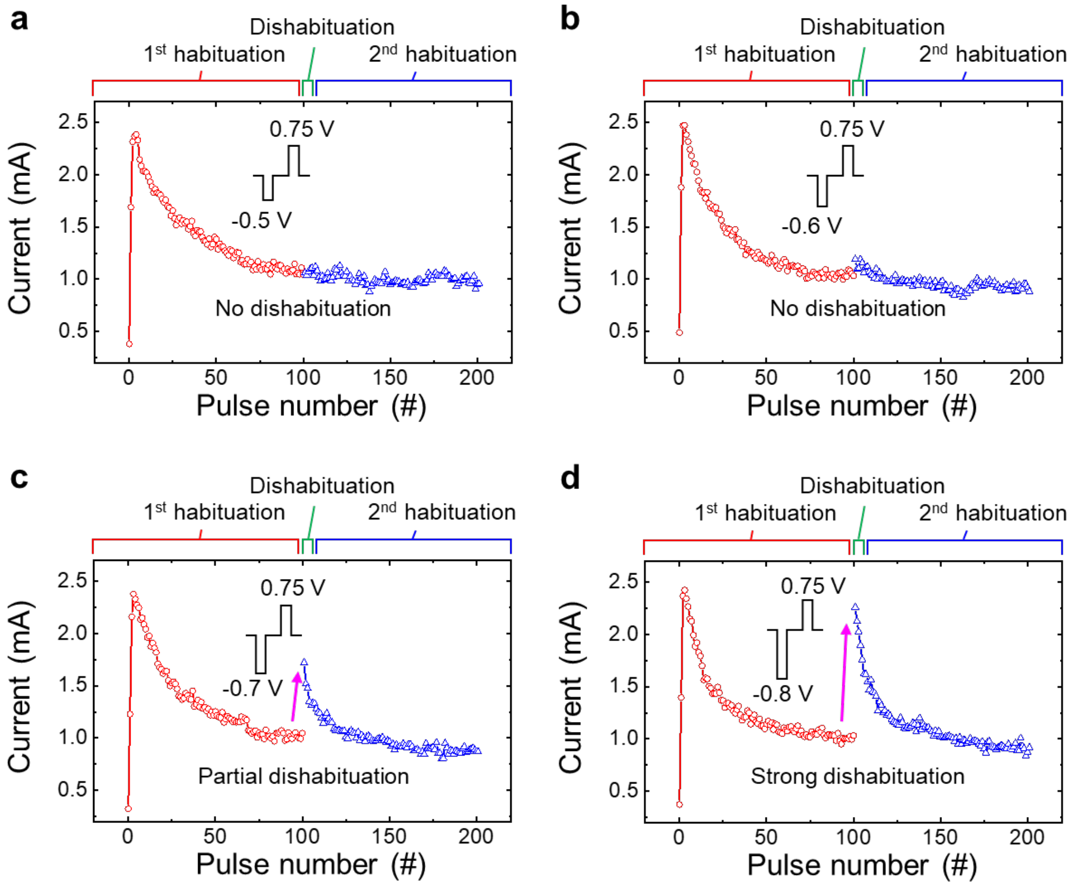

Supplementary Figure S25. Effects of the negative voltage pulse amplitude on dishabituation. Dishabituation stimulus with combined voltage pulses was applied to the device after habituation. **a.** Dishabituation with -0.5 V and 1 μs of negative voltage pulse. **b.** Dishabituation with -0.6 V and 1 μs of negative voltage pulse. **c.** Dishabituation with -0.7 V and 1 μs of negative voltage pulse. **d.** Dishabituation with -0.8 V and 1 μs of negative voltage pulse. As the larger negative voltage pulse amplitude, which represents the strength of the pain stimulus, is applied, the more pronounced dishabituation is observed. In this experiment, positive voltage pulses of 0.75 V and 5 μs and read voltage pulses of 0.3 V and 100 μs were utilized.

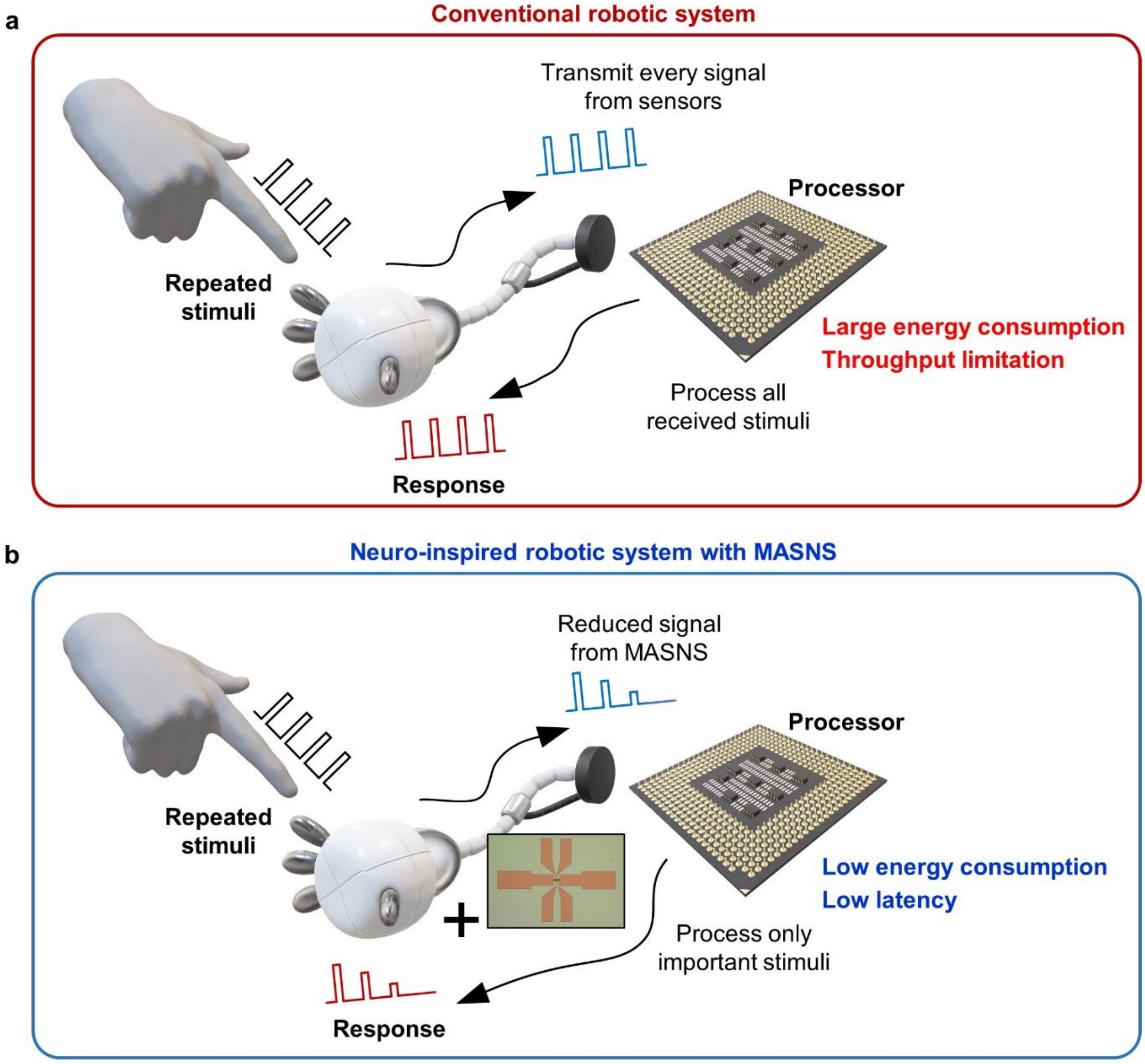

Supplementary Figure S26. Illustrations of the effectiveness of the MASNS for robotic systems. a. Conventional robotic system without MASNS. In the conventional robotic system, the processor receives all the signals from sensors, resulting in large energy consumption and throughput limitations. **b.** Neuro-inspired robotic system with MASNS. In the robotic system with MASNS, the MASNS with third-order memristor filters out insignificant stimuli. The processor in this system processes only important stimuli. Therefore, the energy efficiency and latency of the system can be improved.

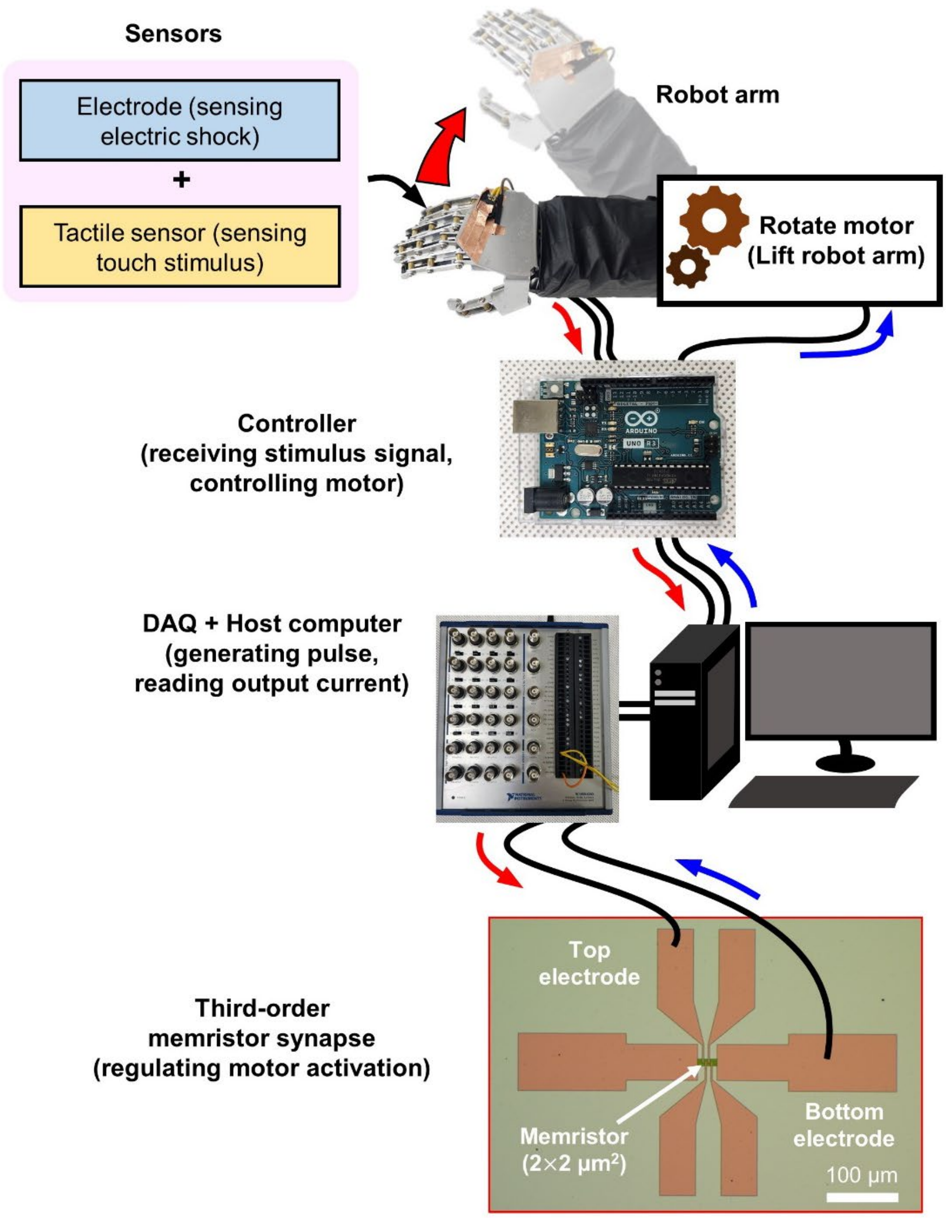

Supplementary Figure S27. Experimental setting of the MASNS-implemented robot arm. The MASNS-implemented robot arm, which senses tactile and electrical stimuli and responds to the stimulus, is built by connecting sensors, a controller (Arduino Uno), a host computer, a data acquisition tool (DAQ), and a memristor. When the sensor receives a stimulus, the signal is transferred to the host computer and the DAQ generates a corresponding voltage pulse to the received stimulus followed by a read pulse. The memristor conductance is measured and the rotating angle of the rotating motor in the robot arm is determined based on the measured conductance. The relationship between the memristor conductance and rotating angle is represented as follows.

$$Rotating\ angle\ (\theta) = Min(90°, \varphi \times (\sigma - \sigma_{\mathrm{Th}}))$$

The rotating angle (θ) means the moving angle of the robot arm regarding to the applied stimulus, φ means a unit angle of the rotation (0.6° for low-order memristor case and 0.15° for third-order memristor case), σ means the conductance of the memristor after applying a stimulus, and σ_{Th} means the threshold conductance of the motor neuron. The maximum rotating angle of the robot arm is fixed as 90°.

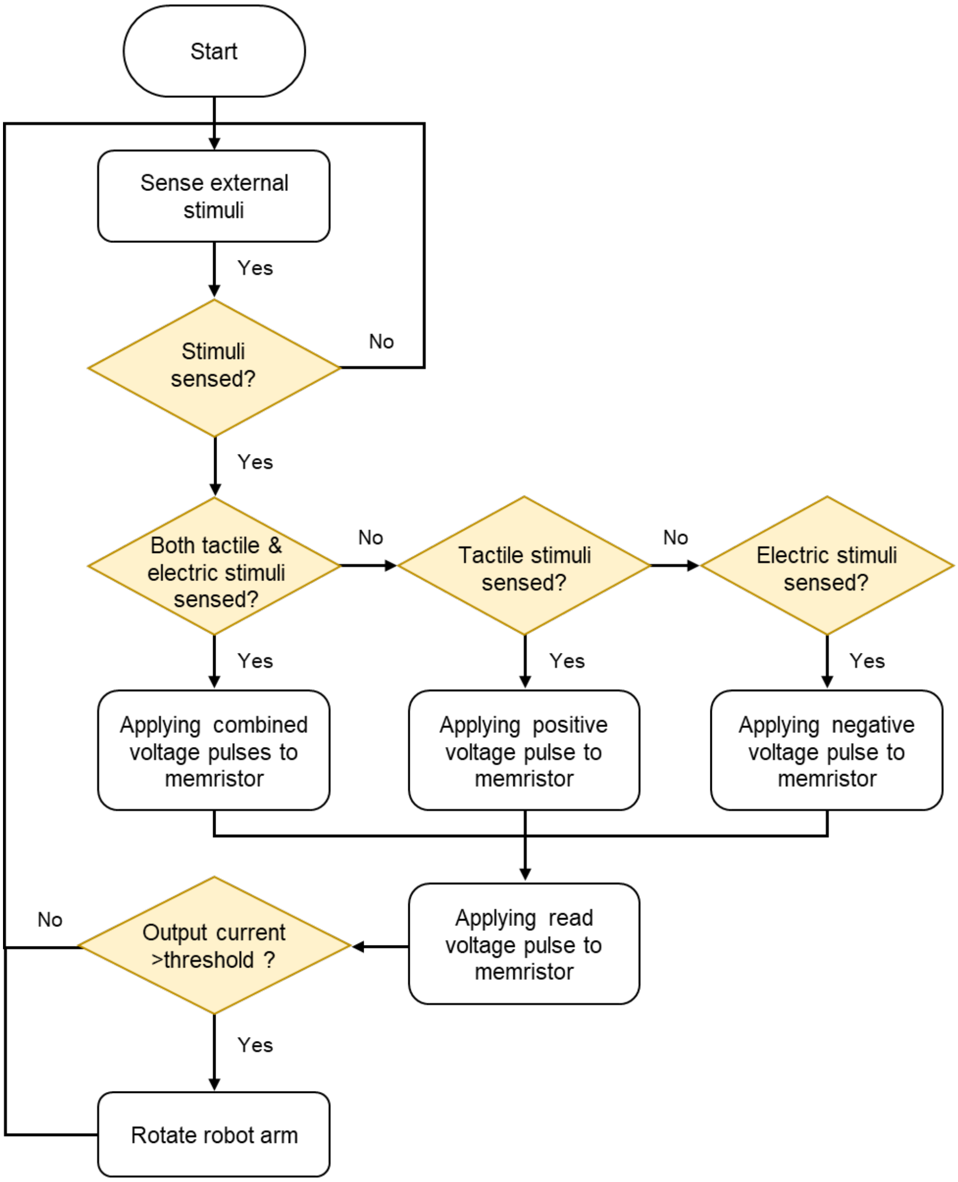

Supplementary Figure S28. Operation algorithm of the robot arm system. The tactile and electric sensors were attached on the robot arm to sense applied stimuli. When tactile or electric (pain) stimuli is applied, the sensors generate output voltage which is received by the Arduino controller. Then, the Arduino controller sends a signal to the DAQ informing which type of stimuli (tactile, pain, or both) is applied. According to the type of applied stimuli, the DAQ applies corresponding voltage pulse, which is a pre-synaptic spike in the biological counterpart, to the third-order memristor. The applied voltage pulse modulates the conductance of the memristor, thereby updating the synaptic strength properly. Next, the DAQ applies a read voltage pulse to the memristor to generate output current, which is a post-synaptic spike in the biological counterpart. Finally, according to the amplitude of this output current and the threshold current, the host PC calculates the amplitude of the response and controls the Arduino controller to operates the rotate motor.

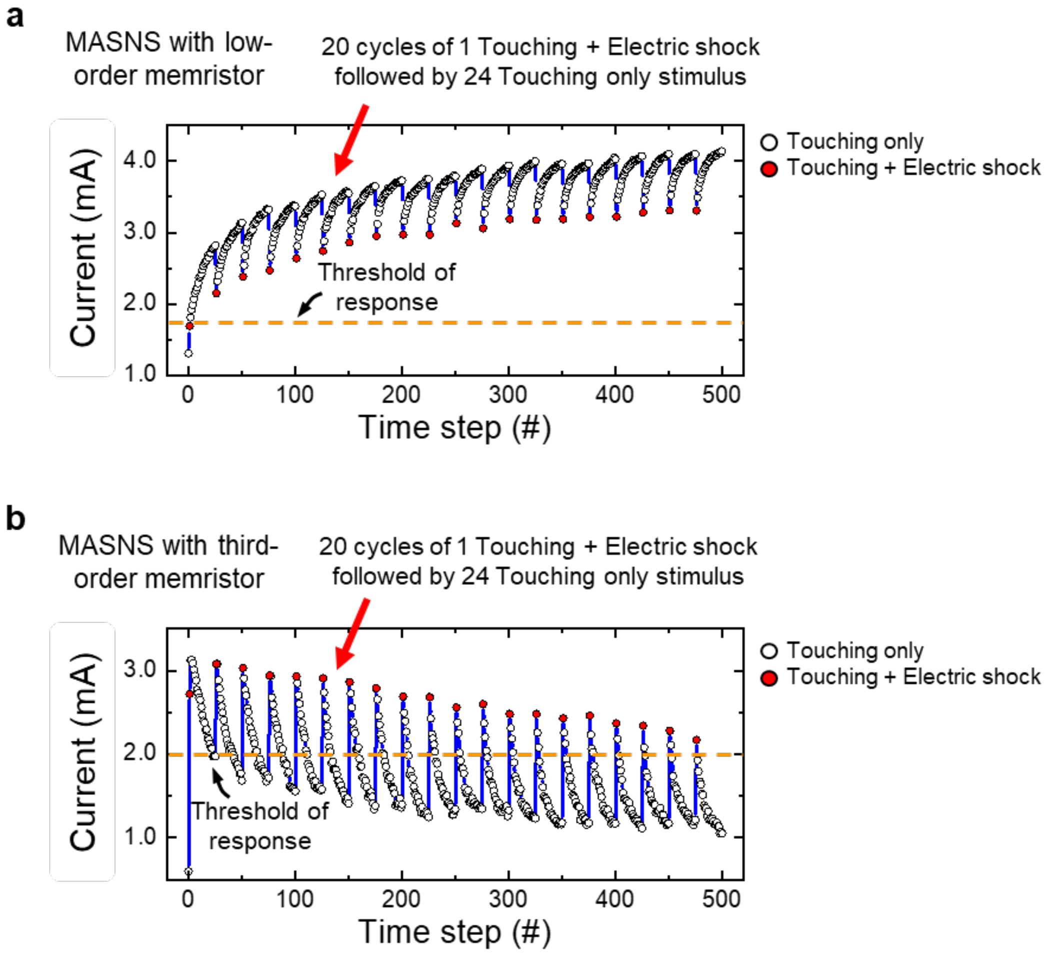

Supplementary Figure S29. The conductance curves of the MASNS-implemented robot arm for 500 stimuli. The conductance of the memristor is measured while a total of 500 stimuli are applied. In the experiment, 20 cycles of voltage pulse trains, where the single voltage pulse train consists of one touching+electric shock case pulse and 24 touching only case pulse, are applied to the low-order (**a**) and the third-order (**b**) memristors. For the low-order memristor case, the memristor conductance exceeds the threshold (orange line) after applying two touching only case stimuli, which means that the low-order memristor wastes unnecessary energy to respond to the safe stimulus. The low-order memristor ignores only one safe stimulus among 480 safe stimuli. On the other hand, the conductance of the third-order memristor ignores 341 among 480 safe stimuli by habituation, while it sensitively responds to the touching+electric shock case stimulus by sensitization. Therefore, the third-order memristor saves energy by ignoring safe stimuli. Furthermore, the third-order memristor exhibited long-term habituation similar to that observed in the biological SNS, where there is an overall decrement of synaptic strengths due to repetitive safe input stimuli, proving its similarity to the biological SNS. Here, a positive voltage pulse of 0.75 or 0.8 V and 1 μs is utilized as the touching signal pulse in the low-order and the third-order memristor case, respectively, while a negative voltage pulse (-0.9 V and 1 μs) is utilized for the electric shock signal pulse. A 0.3 V and 100 μs of voltage pulse is used as a read pulse.

Ref.	1	13	2	14	15	16	17	**This work**
Device	TiN/ Li_xSiO_y/Pt	Au/$LiTaO_3$ /Pt	Pt/LLTO/ Pt	Pt/SNO/Pt	Pt/NiO/Pd	W/HfO_x/ Ti/TiN	Al/HfO_x/ IGZO/Au	**TiN/HfO_2/TiO_x/ Ti/TiN**
CMOS Compatibility	No	No	No	No	No	Yes	No	**Yes**
Retention	10,000 s	>1,000 s	<200 s	12 h	20 s	N/A	N/A	**10 years at 440.42 K**
Cycle-to-cycle uniformity	N/A	N/A	N/A	N/A	2%	N/A	N/A	**LRS: 3.9% HRS: 21.1%**
Device-to-device uniformity	N/A	N/A	N/A	N/A	N/A	N/A	N/A	**LRS: 7% HRS: 27%**
Switching speed	100 ns	1 µs	0.4 s	200 s	0.5 s	1 ms	50 ms	**250 ns**
Habituation	Yes	Yes	Yes	Yes	Yes	Yes	Yes	**Yes**
Dishabituation	Yes	No	Yes	No	No	Yes	No	**Yes**
Habituation of dishabituation	Yes	No	Yes	No	No	No	No	**Yes**
Stimulus intensity dependence	Yes	Yes	Yes	No	Yes	Yes	Yes	**Yes**
Stimulus frequency dependence	Yes	Yes	Yes	No	Yes	Yes	Yes	**Yes**
Potentiation of habituation	Yes	No	No	No	No	No	No	**Yes**
Spontaneous recovery	Yes	No	Yes	Yes	Yes	No	Yes	**Yes**
Application	Robot navigation (Simul.)	N/A	N/A	Spiking neural network (Simul.)	Homeostatic learning (Simul.)	N/A	N/A	**Neuro-inspired robot arm control (Exp.)**

Supplementary Table S1. Comparison with various memristors emulating habituation characteristics. To demonstrate advantages of the third-order memristor for emulating habituation characteristics, several key characteristics are compared. For utilizing a MASNS in robotic applications, non-volatile memory, good uniformity, fast switching speed, as well as ability to emulate various habituation characteristics are required. The comparison reveals the third-order switching complexity enables non-volatile habituation state and various habituation characteristics, while CMOS-compatible HfO_2-based resistive switching offers good uniformity and fast switching speeds.

Set pulse conditions	0.5-1 μs	1-3 μs	3-5 μs
**0.6-0.7 V**	No switching	Monotonic update	Monotonic update
**0.7-0.8 V**	Monotonic update	Monotonic update or Habituation	Habituation
**0.8-0.9 V**	Habituation	Habituation	Habituation

Supplementary Table S2. Effects of set pulse conditions on switching characteristics of the device. The device exhibited various switching characteristics, such as no switching, monotonic conductance update, or habituation characteristics, depending on the pulse conditions. Notably, when the pulse amplitude was 0.7-0.8 V and the width was 1-3 μs, the device exhibited either monotonic update or habituation, depending on the specific pulse amplitude, width, or interval. The table demonstrates that the switching characteristics of the device can be finely tuned by adjusting the pulse conditions.

Ref.		Power (mW)	Pulse width for each processing (ms)
18 (Habituation and sensitization)		12.06 (average)	~2
19 (Associative learning)		7.78 (average)	2
This work	Habituation	**3.94**	**0.001**
	Sensitization (Dishabituation)	**4.63**	**0.002**
	Standby (Retaining synaptic state)	**N/A**	**N/A**

Supplementary Table S3. Comparisons of power consumption and processing time of the third-order memristor and circuit-based systems. For the circuit-based systems, multiple circuit modules are required for emulating synaptic functions such as non-associative or associative learnings. The need of multiple circuit modules and complex processing steps increase the power consumption and processing time. On the contrary, the third-order memristor exhibits habituation and sensitization characteristics based on its non-monotonic conductance update characteristics, without the need of multiple peripheral circuitries. The absence of complex peripheral circuitries and multiple processing steps results in the reduced power consumption and rapid processing time. Furthermore, due to the non-volatile memory characteristics of the third-order memristor, the synaptic state can be maintained during the standby or off state without the need of power-consuming refresh operations.

Supplementary Note S1

Synaptic behaviors associated with habituation

There are various representative synaptic behaviors relevant to habituation[20]. The detailed explanations regarding each synaptic behavior emulated by the third-order memristor are as follows:

1) Dishabituation

In biological sensory nervous systems, applying different stimuli leads to a recovery of the reduced response to the original state[20]. This recovery from a habituated state to a sensitized state is called dishabituation. Dishabituation is one of the most significant synaptic behaviors that help distinguish whether a reduced response is due to habituation rather than motor neuron fatigue. To test the dishabituation characteristic of the third-order memristor, we first applied repetitive positive voltage pulses corresponding to tactile stimuli to the device, resulting in the habituation state (see Supplementary Fig. S19a). After the habituation, combined negative and positive voltage pulses corresponding to pain and tactile stimuli, respectively, were applied to the device in the habituation state. Notably, the application of this different stimulus, or combined voltage pulse, caused the device conductance to a low-resistance state (LRS), mirroring the biological dishabituation phenomenon. These results demonstrate that the device exhibits dishabituation, with the conductance of the habituated device recovering upon the presentation of different stimuli.

2) Habituation of dishabituation

In biological sensory nervous systems, repeated application of dishabituating stimuli—which initially disrupt habituation—leads to a gradual reduction in response. This phenomenon is known as the habituation of dishabituation. To observe this habituation of dishabituation in the third-order memristor, we applied consecutive dishabituating stimuli of the combined voltage pulses after the habituation process, as shown in Supplementary Fig. S19b. Initially, the dishabituating pulses caused a significant increase in the device conductance, resulting in dishabituation. However, as these dishabituating pulses were repeatedly applied, the conductance of the device gradually decreased, demonstrating the habituation of dishabituation.

3) Stimulus frequency dependency

When the frequency of stimulation is higher, the more rapid and pronounced habituation is observed in the biological sensory nervous systems, and this phenomenon is called stimulus frequency dependency. To assess the effect of frequency on habituation in the third-order memristor, we applied two positive voltage set pulses with different pulse intervals, followed by a read pulse. This pulse train was repeated 50 times for each interval, and the resulting conductance update behavior was compared by analyzing the final output current (see Supplementary Fig. S19c). As shown in Supplementary Fig. S19d, the device exhibited faster habituation and lower final output current for shorter pulse interval cases. This frequency dependency originates from the thermal effect of the device, where a shorter pulse interval generates larger Joule heat with less heat dissipation. The observed variation in final output current with different habituation behaviors demonstrates the stimulus frequency dependency of the developed device.

4) Stimulus intensity dependency

When the stimulus intensity is lower, the more rapid and pronounced habituation is observed in the biological sensory nervous systems, and this phenomenon is called stimulus intensity dependency. To examine the stimulus intensity dependency of the device, we applied consecutive 100 positive set voltage pulses with different amplitudes from 0.75 to 1.1 V and measured the output current using read pulses (see Supplementary Fig. S19e). As shown in the Supplementary Figs. S19e and S19f, the device showed pronounced habituation with lower final output current at a pulse amplitude of 0.75 V. However, when voltage pulses with higher amplitude were applied, the increased voltage induced the formation of a thicker filament, resulting in a higher final output current. These results demonstrate that the device exhibits stimulus intensity dependency similar to the biological counterpart.

5) Spontaneous recovery

Spontaneous recovery refers to the recovery of a response after a period of stimulus absence after habituation. In several previous studies, spontaneous recovery has been realized in volatile memory characteristics of devices, where trained information naturally fades due to the short retention of the memory. The non-volatile memory of the third-order memristor might be considered a discrepancy compared to biological synapses. While volatile memory can simulate spontaneous recovery, it is challenging to develop non-volatile or long-term memory of trained information in such volatile memory devices. In contrast, the non-volatile memory offers advantages in terms of reliability and energy-efficiency for robotic applications, as short-term memory devices would lose trained information over time or when powered off, making them unsuitable for such applications.

Furthermore, spontaneous recovery in non-volatile devices can be simulated by applying periodic weak reset pulses, which we termed pseudo spontaneous recovery, as shown in Supplementary Fig. S19g. To emulate the spontaneous recovery using periodic weak reset pulses, we first applied consecutive 100 set pulses for habituation. Afterward, set pulses were withheld for 100 seconds, and weak negative voltage reset pulses were applied to the device every 10 seconds during the stimulus absence. The partial recovery of the device conductance could be observed after the stimulus withholding period, resembling the spontaneous recovery in biological systems. These results demonstrate that the device can simulate spontaneous recovery through the use of periodic reset pulses, if such characteristics are required by the application system. However, it is noteworthy that, since various robotic applications use batteries and have limited power sources, it is crucial to maintain stored information without decaying when powered off, rather than accurately mimic biological synaptic behaviors.

6) Potentiation of habituation

Potentiation of habituation refers to the phenomenon that the rate or degree of habituation increases with repeated series of stimuli, when periods of spontaneous recovery are alternated between stimulus series. To test the potentiation of habituation in the developed device, we applied two series consecutive set pulses, with 100 seconds of stimulus withholding between each habituation phase. During the stimulus withholding, the periodic weak reset pulses were applied to the device every 10 seconds to induce pseudo spontaneous recovery. As shown in Supplementary Fig. S19h, faster habituation was observed after 100 seconds of pseudo

spontaneous recovery. The results prove that the device could emulate potentiation of habituation, based on pseudo spontaneous recovery with periodic reset pulses.

Supplementary References:

1. Wu, Z. *et al.* A habituation sensory nervous system with memristors. *Adv. Mater.* **32**, 2004398 (2020).
2. Shi, T., Wu, J. F., Liu, Y., Yang, R. & Guo, X. Behavioral plasticity emulated with lithium lanthanum titanate-based memristive devices: Habituation. *Adv. Electron. Mater.* **3**, 1700046 (2017).
3. Zhao, B., Xiao, M., Shen, D. & Zhou, Y. N. Heterogeneous stimuli induced nonassociative learning behavior in ZnO nanowire memristor. *Nanotechnology* **31**, 125201 (2020).
4. Lee, T. J. *et al.* Realization of an artificial visual nervous system using an integrated optoelectronic device array. *Adv. Mater.* **33**, 2105485 (2021).
5. Zhang, Z. *et al.* Neuromorphic learning with Mott insulator NiO. *Proc. Natl. Acad. Sci.* **118**, e2017239118 (2021).
6. Yao, P. *et al.* Fully hardware-implemented memristor convolutional neural network. *Nature* **577**, 641–646 (2020).
7. Rao, M. *et al.* Thousands of conductance levels in memristors integrated on CMOS. *Nature* **615**, 823–829 (2023).
8. Park, S. O., Jeong, H., Park, J., Bae, J. & Choi, S. Experimental demonstration of highly reliable dynamic memristor for artificial neuron and neuromorphic computing. *Nat. Commun.* **13**, 2888 (2022).
9. Kim, S. *et al.* Experimental demonstration of a second-order memristor and its ability to biorealistically implement synaptic plasticity. *Nano Lett.* **15**, 2203–2211 (2015).
10. Kumar, S., Wang, X., Strachan, J. P., Yang, Y. & Lu, W. D. Dynamical memristors for higher-complexity neuromorphic computing. *Nat. Rev. Mater.* **7**, 575–591 (2022).
11. Jiang, H., Li, C. & Xia, Q. Ta/HfO_2 memristors: From device physics to neural networks. *Japanese Journal of Applied Physics* **61**, SM0802 (2022).
12. Peng, W. C. *et al.* Tunability of p- and n-channel TiO_x thin film transistors. *Sci. Rep.* **8**, 9255 (2018).
13. Li, X. *et al.* Implementation of habituation on single ferroelectric memristor. *Appl. Phys. Lett.* **122**, 183505 (2023).
14. Zuo, F. *et al.* Habituation based synaptic plasticity and organismic learning in a quantum perovskite. *Nat. Commun.* **8**, 240 (2017).
15. Mondal, S. *et al.* All-electric nonassociative learning in nickel oxide. *Adv. Intell. Syst.* **4**, 2200069 (2022).
16. Yang, X. *et al.* Nonassociative learning implementation by a single memristor-based multi-terminal synaptic device. *Nanoscale* **8**, 18897–18904 (2016).
17. Jiang, R., Ma, P., Han, Z. & Du, X. Habituation/Fatigue behavior of a synapse memristor based on IGZO-HfO_2 thin film. *Sci. Rep.* **7**, 9354 (2017).
18. Hong, Q. *et al.* Memristive circuit implementation of biological nonassociative learning mechanism and its applications. *IEEE Transactions on Biomedical Circuit and Systems* **14**, 1036-1050 (2020).
19. Zhang, Y. *et al.* The framework and memristive circuit design for multisensory mutual associative memory networks. *IEEE Transactions on Cybernetics* **53**, 7844-7857 (2023).
20. Rankin, C. H. *et al.* Habituation revisited: An updated and revised description of the behavioral characteristics of habituation. *Neurobiol. Learn. Mem.* **92**, 135–138 (2009).
