# Supplementary material for: Experimental demonstration of third-order memristor-based artificial sensory nervous system for neuro-inspired robotics
Source: Nat Commun. 2025 Jul 1;16:5754. doi: 10.1038/s41467-025-60818-x (PMC12215477; doi:10.1038/s41467-025-60818-x)
Supplement: Supplementary file 2 — Description of Additional Supplementary Files [file 41467_2025_60818_MOESM2_ESM.pdf]

Description of Additional Supplementary Files

Supplementary Movie 1. The response of the robot arm with a conventional low-order memristor-based MASNS according to the applied stimuli.

Supplementary Movie 2. The response of the robot arm with the third-order memristor-based MASNS according to the applied stimuli.
